# DDX56 transcriptionally activates MIST1 to facilitate tumorigenesis of HCC through PTEN-AKT signaling

**DOI:** 10.7150/thno.72471

**Published:** 2022-08-15

**Authors:** Hongzhong Zhou, Yiqun Du, Xiafei Wei, Chunli Song, Jianning Song, Nanson Xu, Weihong Huang, Lichan Chen, Fuwen Yao, Duanming Du, Chuanghua Qiu, Lihong Zhong, Yuchen Liu, Dayong Gu, Jin Wang, Yong Xu

**Affiliations:** 1 Department of Laboratory Medicine , Shenzhen Institute of Translational Medicine, The First Affiliated Hospital of Shenzhen University, Shenzhen Second People's Hospital, Shenzhen University, Shenzhen, China.; 2 Institute for Hepatology, National Clinical Research Center for Infectious Disease, Shenzhen Third People's Hospital, Southern University of Science and Technology, Shenzhen, China.; 3 Clinical Laboratory Medicine Center, Shenzhen Hospital, Southern Medical University, Shenzhen, China.; 4 Sun Yat -sen University Cancer Center, State key laboratory of Oncology in South China, Collaborative Innovation Center for Cancer Medicine.; 5 Department of Pathology, Guangdong Provincial People's Hospital, Guangdong Academy of Medical Sciences.; 6 Guangzhou Medical University, Guangzhou, China.; 7 Department of Interventional Therapy, The First Affiliated Hospital of Shenzhen University, Health Science Center, Shenzhen Second People's Hospital, Shenzhen, China.; 8 Shenzhen Institute of Advanced Technology, Chinese Academy of Sciences, Shenzhen, China.

**Keywords:** Hepatocellular carcinoma, Transcription, Zinc finger E-box binding homeobox 1, Proliferation, DEAD-Box 56

## Abstract

**Rationale**: Hepatocellular carcinoma (HCC) is a primary malignancy of the liver that is the leading cause of cancer-related mortality worldwide. However, genetic alterations and mechanisms underlying HCC development remain unclear.

**Methods:** Tissue specimens were used to evaluate the expression of DEAD-Box 56 (DDX56) to determine its prognostic value. Colony formation, CCK8, and EdU-labelling assays were performed to assess the effects of DDX56 on HCC proliferation. The *in vivo* role of DDX56 was evaluated using mouse orthotopic liver xenograft and subcutaneous xenograft tumor models. Dual-luciferase reporter, chromatin immunoprecipitation, and electrophoretic mobility shift assays were performed to examine the effect of DDX56 on the MIST1 promoter.

**Results:** DDX56 expression in HCC tissues was elevated and this increase was strongly correlated with poor prognoses for HCC patients. Functionally, DDX56 promoted HCC cell proliferation both *in vitro* and *in vivo*, while mechanistically interacting with MECOM to promote HCC proliferation by mono-methylating H3K9 (H3K9me1) on the MIST1 promoter, leading to enhanced MIST1 transcription and subsequent regulation of the PTEN/AKT signaling pathway, which promotes HCC proliferation. More importantly, the PTEN agonist, Oroxin B (OB), blocked the DDX56-mediated PTEN-AKT signaling pathway, suggesting that treating HCC patients with OB may be beneficial as a therapeutic intervention. Furthermore, we observed that ZEB1 bound to DDX56 and transcriptionally activated DDX56, leading to HCC tumorigenesis.

**Conclusions:** Our results indicated that the ZEB1-DDX56-MIST1 axis played a vital role in sustaining the malignant progression of HCC and identified DDX56 as a potential therapeutic target in HCC tumorigenesis.

## Introduction

Hepatocellular carcinoma (HCC) is one of the most common malignant tumors and the second leading cause of cancer-related deaths worldwide 1. Significant risk factors for HCC include viral hepatitis (HBV and HCV), non-alcoholic liver steatohepatitis/non-alcoholic fatty liver disease, and alcoholic liver disease 2. Despite significant technological advances, such as liver transplantation, surgical procedures, and chemotherapy, that have been made during recent decades, the 5-year survival rate of HCC remains at approximately 18%, largely due to its insidious and aggressive nature 1. Accordingly, elucidation of the molecular pathogenesis of HCC may lead to more specific therapeutic targets against cancer being revealed.

DEAD-Box 56 (DDX56) is a member of the RNA helicase DDX family. The DDX family is involved in nearly all aspects of RNA metabolism, including pre-mRNA splicing, transcription, miRNA processing, and translation. Evidence indicates that dysregulation of DDX family proteins, such as DDX3 3, DDX5 4, DDX17 5, DDX20 6, and DDX39 7, is frequently associated with poor prognoses for HCC patients. Nevertheless, the function of DDX56 in HCC remains obscure. It has been shown that DDX56 dysregulation is associated with squamous cell lung carcinoma 8, glioblastoma 9, osteosarcoma 10, and colorectal cancer 11, suggesting that DDX56 may play a critical role in cancer development and progression. Thus, deciphering the relationship between DDX56 expression and its impact on the prognosis for HCC patients as well as its direct effect on hepatocarcinogenesis may reveal novel diagnostic and therapeutic targets.

Muscle intestine stomach expression 1 (MIST1), also known as BHLHA15, belongs to the basic helix-loop-helix (bHLH) superfamily, which plays a dual role in cancer development. MIST1 is regarded as a tumor promoter in multiple cancers, including cervical cancer 12, colon cancer 13, acinic cell carcinoma 14, and cutaneous malignant melanoma 15. However, previous studies have shown that upregulation of MIST1 reverses epithelial-mesenchymal transition (EMT) and attenuates the tumorigenicity of pancreatic cancer and gastric cancer 16, 17. Thus, the relationship between MIST1 and cancer remains controversial. More importantly, the role played by MIST1 in HCC, as well as that played by DDX56 in MIST1 regulation, remain unclear.

In this study, we found that DDX56 was significantly upregulated in the tissues of HCC patients and that such upregulation was strongly correlated with poor prognoses for these patients. Moreover, whereas DDX56 overexpression promoted HCC proliferation *in vitro* and* in vivo,* its knockdown inhibited such proliferation. Mechanistically, DDX56 overexpression enhanced MIST1 transcription by recruiting the MDS1 and EVI1 complex locus (MECOM), also known as EVI-1, to the promoter of MIST1, thereby inducing the PTEN-AKT signaling pathway to promote HCC proliferation. Crucially, the pharmacological intervention of PTEN function using Oroxin B, a PTEN agonist, significantly blocked the DDX56-mediated PTEN-AKT signaling pathway, suggesting a rationale for the potential treatment of HCC patients with Oroxin B. However, the mechanism underlying the dysregulation of DDX56 remains elusive.

Zinc finger E-box-binding homeobox 1 (ZEB1) has been identified as a transcription factor which binds to E2-box-like CACCT(G), located in the promoter regions of target genes 18. Overexpression of ZEB1 leads to the progression of multiple cancers, including gastric carcinoma, pancreatic cancer, and colorectal cancer 19. Studies conducted by us as well as others have indicated that high ZEB1 expression is significantly correlated with poor prognoses for HCC patients 20. However, whether ZEB1 upregulates DDX56 and consequently promotes HCC tumorigenesis remains largely unknown. In this study, we found that ZEB1 directly activates DDX56 transcription and contributes to the proliferation of HCC to a great extent. Overall, our findings revealed the molecular mechanisms underlying the ZEB1-DDX56-MIST1 axis in hepatocarcinogenesis, thereby providing a potential therapeutic target for HCC treatment.

## Methods

### Cell culture and sample collection

Huh-7, PLC/PRF/5, MHCC97H, Hep3B, and Immortalized human liver cell line (L02) cells were obtained from Jennio Biotech Co. Ltd. (Guangzhou, China). The cells were cultured in Dulbecco's modified Eagle's medium (DMEM; Gibco) supplemented with 10% fetal bovine serum (FBS; Gibco) at 37 °C and 5% CO_2_. The cells were authenticated via short tandem repeat analysis every 6 months. Human HCC tissues and paired non-tumor adjacent tissues were obtained from the First Affiliated Hospital of Shenzhen University (Shenzhen, China). The ethics application was approved by the Ethics Committee (application number KS20190903002) of the First Affiliated Hospital of Shenzhen University. Written informed consent was obtained from all the patients.

### Immunohistochemistry

An HCC tissue array, containing 32 paraffin-embedded primary HCC specimens and paired non-tumor adjacent tissues, was obtained from Servicebio (Wuhan, China). Immunohistochemistry (IHC) staining was performed according to standard protocols. Briefly, primary antibodies against DDX56 (GTX115551, GeneTex, Texas, USA) and MIST1 (NBP2-22478, Novus, USA) were incubated at 4 °C overnight. Evaluation of immunohistochemical staining was based on a histochemical scoring (H-score) assessment which incorporated both staining intensity and the percentage of stained cells at each intensity level. Staining intensities were scored as 0 (no evidence of staining), 1 (weak staining), 2 (moderate staining), and 3 (strong staining). The total number of cells per field of view and the number of positively stained cells at each intensity level were counted. The percentage of stained cells at each intensity level varied from 0% to 100%. The H-score was calculated as follows: H-score = (% of cells stained at intensity category 1 × 1) + (% of cells stained at intensity category 2 × 2) + (% of cells stained at intensity category 3 × 3). An H-score between 0 and 300 was obtained for each staining.

### Real-time polymerase chain reaction (RT-PCR)

The total RNA content from HCC cells and tissues was extracted using the AG RNAex Pro Reagent (AG21102, Accurate Biotechnology, Hunan, China). One microgram of RNA was used for reverse transcription, and first-strand cDNA was synthesized using a TransScript First-Stand cDNA Synthesis kit (Tiangen, Beijing, China) according to the manufacturer's instructions. GAPDH was used as the control. Real-time PCR was performed using a SYBR Green Kit (Bio-Rad, USA). Primers used in this study are listed (Table S1).

### Western blot

The total protein content was obtained from the cell lines or tissues via lysis in a radioimmunoprecipitation assay buffer containing a protease inhibitor cocktail mini-tablet (Roche, Basel, Switzerland). The proteins were separated using SDS-PAGE and then transferred to a polyvinylidene difluoride (PVDF) membrane (Millipore, USA). The membrane was incubated in blocking buffer (TBST containing 5% non-fat milk) and then incubated with corresponding antibodies. After washing with TBST thrice, the membranes were incubated with the secondary antibody for 2 h at room temperature and visualized using ECL (Millipore). Antibodies used in this study are listed (Table S2).

### Plasmids and short hairpin RNA

The plasmids, including DDX56, MIST1, MECOM and PTEN, were synthesized by GeneChem (Shanghai, China). These plasmids were transfected into HCC cells using Lipofectamine 3000 (Invitrogen, USA). Lentiviral short hairpin RNA (shRNA) targeting DDX56, MIST1, and MECOM were cloned into lentiviral vectors. HCC cells were infected with lentiviral particles as previously described. After 48 or 72 h, HCC cells were harvested for use in further experiments. The shRNA sequences are listed (Table S3).

### Cell viability assays

The HCC cells were seeded in 96-well plates at a density of 2×10^3^ cells/well and examined at 0, 24, 48, 72, and 96 h. Cell viability was measured via a Cell Counting kit-8 (CCK-8) assay (MCE, Shanghai, China). A total of 100 μL of CCK8 solution (10 μL CCK8+90 μL medium) was added to each well and incubated for 2 h. Absorbance was measured at 450 nm using a Nanodrop 2000 spectrophotometer.

### Colony formation assays

HCC cells were seeded in 6-well plates at a density of 1×10^3^/well for 2 weeks and fixed with methanol. After staining with 0.05% crystal violet (Servicebio) for 30 min, colonies were counted under a microscope.

### EdU labelling and immunofluorescence assay

An EdU labelling assay was performed using an Apollo®488 Imaging Kit (RiboBio, Guangzhou, China) according to the manufacturer's protocol. Briefly, HCC cells were cultured in 48-well plates and incubated with 50 μM 5-ethynyl-2´-deoxyuridine (EdU) for 2 h. The cells were then fixed in 4% paraformaldehyde and permeabilized with 0.5% Triton X-100 for 10 min. The slides were stained with Apollo®488 at RT for 30 min, and subsequently stained with DAPI for 10 min. EdU-positive cells were counted using ImageJ software.

### Dual-luciferase reporter assay

Briefly, the promoter sequences of MIST1 were cloned into a pGL3-promoter vector. After the HCC cells were seeded in 6-well plates for 48 h, luciferase reporter plasmids and 10 ng of pRL-TK Renilla plasmids were co-transfected into HCC cells using Lipofectamine 3000 according to the manufacturer's instructions. Luciferase and Renilla signals were detected 48 h after transfection of HCC cells using a Dual-Luciferase Reporter Assay Kit (E1910, Promega, USA). Primers used for promoter construction are listed (Table S1).

### Chromatin immunoprecipitation assay

Briefly, HCC cells were grown to 80-90% confluence on a 10 cm dish and treated with 1% formaldehyde for 10 min. Subsequently, cell lysates were sonicated to generate DNA fragments. After removing cell debris, the supernatant containing chromatin was immunoprecipitated overnight at 4 °C using anti-DDX56, anti-ZEB1, anti-MECOM, anti-H3K9me1, anti-MIST1, or anti-IgG antibodies (a negative control, Millipore). Then, DNA-protein complexes were pulled down with magnetic beads and subsequently eluted from the beads using an elution buffer. Enrichment of specific DNA fragments was examined using RT-PCR. The products were loaded onto a 1.5% agarose gel and subjected to electrophoresis. All antibodies used in this study are listed (Table S2).

### Electrophoretic mobility shift assay

Recombinant human His-DDX56 protein (N-terminal His-Tag, GTX68554-pro) was purchased from GeneTex. Fragments of MIST1 promoters were synthesized and labelled with biotin by HuaDa Gene Technology Co., Ltd (Shenzhen, China) at the 5´ end. Electrophoretic mobility shift assay (EMSA) analyses were performed using a LightShift^®^ Chemiluminescent EMSA Kit (#20148; Thermo Scientific, MA, USA). Briefly, the binding reaction mixture (20 μL) contained 2 μL 10×binding buffer, 1 μL 50% glycerol, 1 μL 100 mM MgCl2, 1 µg/μL poly (dI•dC), 1 μL 1% NP-40, and 2 µg purified protein. For the competition assay, a 200-fold excess of the unlabeled fragment was added as competitor DNA. For the supershift assay, 3 µg DDX56 antibody (UM800049, Origene, USA) was added and the mixture was incubated at room temperature for 20 min. Bands were detected using a ChemiDoc^TM^ MP Imaging System (Bio-Rad). Probe sequences used in the EMSA assay are listed (Table S1).

### Immunofluorescence

HCC cells were fixed with 4% formaldehyde for 10 min and permeabilized with 0.1% TritonX-100 for 10 min. After routine washing with PBS containing 0.1% TritonX-100, cells were incubated with anti-DDX56 (1:400, GeneTex) and anti-MECOM (1:1000, CST, USA) overnight at 4 °C. Coverslips were counterstained with DAPI and mounted for microscopy.

### Tumor xenograft models

Four-to-six-week-old male BALB/c mice were purchased from Weitong Lihua Experimental Animal Technology Co. Ltd. (Beijing, China). Briefly, mouse subcutaneous xenograft tumor models were constructed by subcutaneously injecting 1×10^6^ HCC cells (MHCC97H and PLC/PRF/5) into the flanks of nude mice. Tumor volumes were measured using vernier callipers every 7 d, and the mice were sacrificed 28 d after implantation. The tumors were excised, weighed, photographed, fixed in 4% paraformaldehyde, and paraffin-embedded for immunohistochemical (IHC) analysis. Tumor volume was calculated using the following formula: Volume = (length×width^2^)/2.

To construct mouse orthotopic liver xenograft tumor models, 1×10^6^ MHCC97H cells suspended in 40 μL Matrigel diluted in DMEM (Matrigel: DMEM, 1:1) were injected into the left lung lobes to initiate the development of orthotopic liver tumors. Nude mice were sacrificed six weeks following injection, and their livers removed for examination. All animal experiments were approved by the First Affiliated Hospital of Shenzhen University, and all animal care procedures were performed in compliance with institutional guidelines.

### Data availability

The RNA-seq data are available in the Gene Expression Omnibus (GEO) database under accession numbers GSE14520 and GSE25097. RNA-sequencing data and normalized results were submitted to the GEO database.

### Statistical analysis

All data were statistically analyzed using the GraphPad Prism software (version 8.0). Significant differences between the two groups were evaluated using a two-tailed Student's t test. Correlations between continuous variables were analyzed using Spearman's correlation analysis. Categorical data were evaluated using the chi-squared test. Each experiment was performed in triplicate. Statistical significance was set at* P <* 0.05.

## Results

### DDX56 overexpression is significantly associated with tumor progression in human HCCs as well as poor prognoses for patients

**Figure 1:**
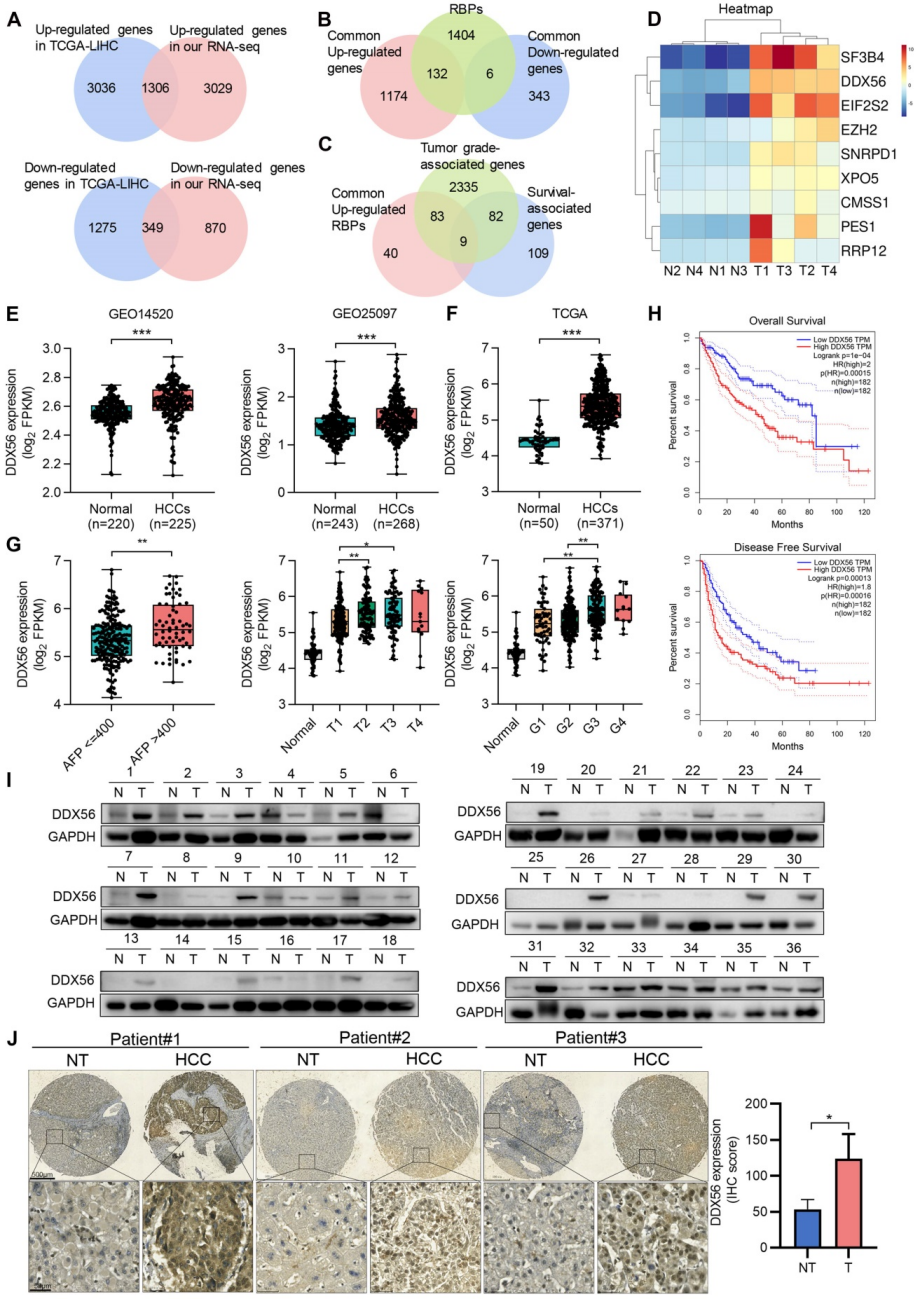
** DDX56 is overexpressed in patients with HCC and correlates with the poor prognosis subtype.** Venn diagrams depicting **(A)** 1306 commonly upregulated genes (Up) and 349 downregulated genes (Down) based on RNA-seq data from TCGA-LIHC and four paired RNA-seq data from our laboratory (|fold change| ≥ 2, *P* < 0.05); **(B)** 132 commonly upregulated and 6 downregulated RNA-binding protein (RBP) genes; **(C)** 9 candidates that were identified based on an analysis of the top 200 survival-associated genes, tumor grade-associated genes, and upregulated RBPs (*P* < 0.05). **(D)** Heatmap showing the expression profiles of these 9 candidate RBPs in four liver cancer tissues and paired adjacent tissues (|fold change| ≥ 2, *P* < 0.05). **(E-F)** Relative mRNA levels of DDX56 in GSE14520 and GSE25097 (***, *P* < 0.0001) (E) and in the TCGA-LIHC database (***, *P* < 0.0001) (F). **(G)** The mRNA level of DDX56 was significantly associated with multiple clinicopathologic parameters, including AFP, T classification, and histologic grade (*, *P* < 0.05, **, *P* < 0.01). **(H)** The DDX56 high expression group showed worse overall survival (OS) and disease-free survival (DFS) in liver cancer patients (OS, *P* = 0.0004; DFS, *P* = 0.00013, Log-rank test). **(I)** Representative western blot images of DDX56 in HCC tissues and the corresponding adjacent normal tissues. **(J)** Representative immunohistochemical staining images of DDX56 from the HCC tissue array obtained from Servicebio (n = 32). Scale bars: 500 µm (top), 50 µm (bottom).

RNA-binding proteins (RBPs) facilitate tumorigenesis in multiple cancers 21. Nevertheless, the role of RBPs in HCC tumorigenesis remains unclear. To explore the role played by RBPs in HCC development, we first generated the gene expression profiles of HCC patients using TCGA-LIHC databases and found 4342 upregulated and 1624 downregulated genes (|fold change| ≥2, *P <* 0.05). To further validate the reliability of public data, we characterized gene expression using RNA-seq analysis of four paired HCC/adjacent tissues (|fold change| ≥2, *P* < 0.05). The two datasets shared 1306 commonly upregulated genes and 349 commonly downregulated genes (Figure 1A). Next, we examined the expression profiles of 1542 RBP genes from the two datasets and found 132 upregulated and 6 downregulated RBP genes (Figure 1B). To gain a better understanding of these 138 differentially expressed RBPs (132 upregulated and 6 downregulated genes) in HCC, Gene Ontology (GO) analysis was performed. GO analysis indicated that these genes were involved in multiple important biological processes, including development, cell communication, cell proliferation, and growth (Figure S1A). To investigate whether these differentially expressed RBPs were associated with HCC progression and poor prognoses, tumor grade and overall survival analyses were performed on all tumor samples. Nine upregulated RBPs were screened as candidate proteins (Figure 1C-D and S1B). Among these RBPs, the roles played by DDX56, CMSS1, and RRP12 in HCC have not yet been explored. To further screen for candidate functional RBPs, we used both GSE14520 and GSE25097 datasets to assess the clinical relevance of the expression of these three RBPs. DDX56 expression was significantly increased in both GSE datasets (GES14520, *P* < 0.0001 and GSE25097, *P* < 0.0001), whereas CMSS1 expression was elevated only in GSE25097 (*P* < 0.0001) but not in GSE14520 (*P* = 0.1228), and RRP12 expression was upregulated only in GSE14520 (*P* < 0.0001), and not in GSE25097 (*P* = 0.5946) (Figure 1E and S1C). Therefore, DDX56 was selected for further studies.

To demonstrate the significantly differential expression of DDX56 in HCC, we analyzed its mRNA expression using the TCGA-LIHC database. Compared with that of normal liver tissues, DDX56 expression in HCC tissues from the TCGA-LIHC database was significantly elevated (Figure 1F). In addition, the Tumor Immune Estimation Resource (TIMER) database showed that the expression levels of DDX56 mRNA were significantly upregulated in most types of solid tumors, including colon adenocarcinoma, esophageal carcinoma, lung adenocarcinoma, and stomach adenocarcinoma (Figure S1D). Moreover, to determine the clinical relevance of DDX56 in HCC, we analyzed the correlation between the mRNA levels of DDX56 and clinicopathological parameters across the TCGA-LIHC database. Specifically, high expression levels of DDX56 were significantly associated with alpha-fetoprotein (AFP) level > 400 (*P* < 0.05), advanced tumor T classification (*P* < 0.05), and histologic grade (*P* < 0.05); (Figure 1G). Consistent with the above results, high DDX56 levels were also consistently associated with shorter overall survival (OS) (*P* = 1e-04) as well as disease-free survival (DFS) (*P* = 0.00013) in liver cancer patients (Figure 1H), suggesting that DDX56 may be considered as an indicator of poor prognosis for HCC.

We then determined DDX56 expression in two independent HCC cohorts. Both mRNA and protein levels of DDX56 were significantly increased in 72 paired HCC patients in cohort 1 (Figure 1I and S1E). Consistent with the results which were obtained from the TCGA database, we found that the expression of DDX56 was correlated with tumor size (*P* = 0.0341), AFP levels (*P* = 0.0486), and HBV positivity (*P* = 0.0496); (Table S4-5). High DDX56 expression was correlated with poor prognoses for HCC patients (*P* = 0.025); (Figure S1F). Moreover, IHC array analysis confirmed that DDX56 expression in HCC tissues was upregulated compared to that in the para-cancerous tissues of cohort 2 (Figure 1J and S1G). Based on immunostaining scores, these patients were randomly divided into high expression (score 2-3) or low expression (score 0-1) groups (Figure S1H). A correlation analysis revealed that high DDX56 expression was significantly associated with tumor size (*P* = 0.0184); (Table S6). Overall, these data indicated that DDX56 overexpression was associated with the malignant progression of HCC.

### DDX56 promotes HCC proliferation *in vitro* and* in vivo*

**Figure 2:**
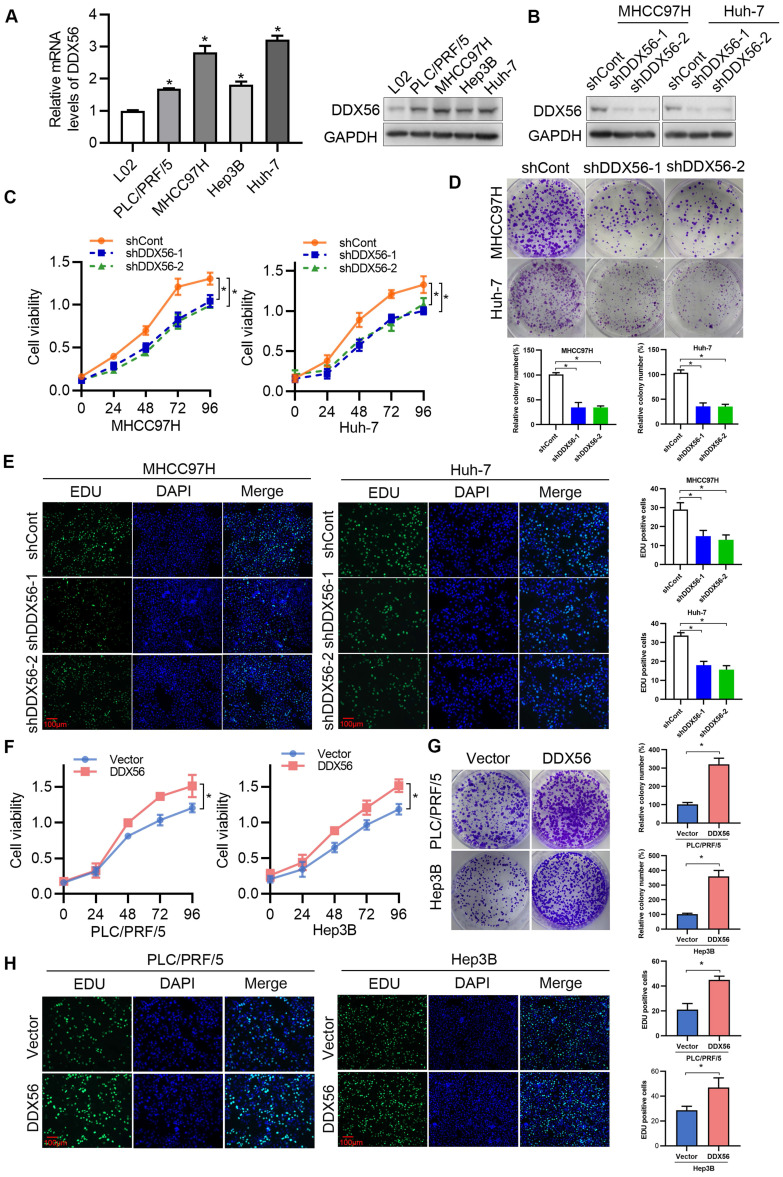
** Effect of DDX56 in promoting HCC cell proliferation* in vitro*. (A)** The mRNA and protein level of DDX56 in HCC cells and immortalized normal liver cells (L02) as determined using RT-PCR and western blot analyses, respectively. **(B)** The lentiviral-mediated shRNA silencing effects of DDX56 in MHCC97H and Huh-7 cells were examined using western blotting. **(C-D)** The effect of DDX56 knockdown on HCC cell proliferation was determined by CCK8 (C) and colony formation assays (D). **(E)** MHCC97H and Huh-7 cells were seeded onto coverslips and DNA synthesis was assessed via EdU immunofluorescence staining. The graph on the right depicts the percentage of EdU-positive nuclei. Scale bars: 100 µm. **(F-H)** Effect of DDX56 gain-of-function on PLC/RPF/5 and Hep3B cell proliferation was determined by CCK8 (F), colony formation assay (G), and EdU assay (H). Scale bars: 100 µm.

To determine the effect of DDX56 on HCC proliferation *in vitro*, endogenous DDX56 mRNA and protein levels in different HCC cell lines were detected using RT-PCR and western blotting, respectively. The results revealed that DDX56 expression in PLC/PRF/5, MHCC97H, Hep3B, and Huh-7 cells was higher than that in immortalized normal liver cells (L02); (Figure 2A). Lentivirus-mediated shRNA was used to silence endogenous DDX56 expression (shDDX56) in MHCC97H and Huh-7 cells (Figure 2B and S2A). DDX56 shRNA-expressing MHCC97H and Huh-7 cells showed reduced cell viability (Figure 2C) and colony formation capacity (Figure 2D) compared with control cells. EdU (5-ethynyl-2´-deoxyuridine) staining of DDX56-shRNA cells revealed that the ratios of EdU-positive nuclei in the former were lower than those of control cells (Figure 2E). Consistent with the results observed in shRNA-DDX56 MHCC97H and Huh-7 cells, DDX56 overexpression promoted cell viability, colony formation capacity, and DNA synthesis in PLC/RFF/5 and Hep3B cells (Figure 2F-H and S2B-C). Together, these gain- and loss-of-function studies indicated that DDX56 plays a critical role in promoting HCC growth *in vitro*.

**Figure 3:**
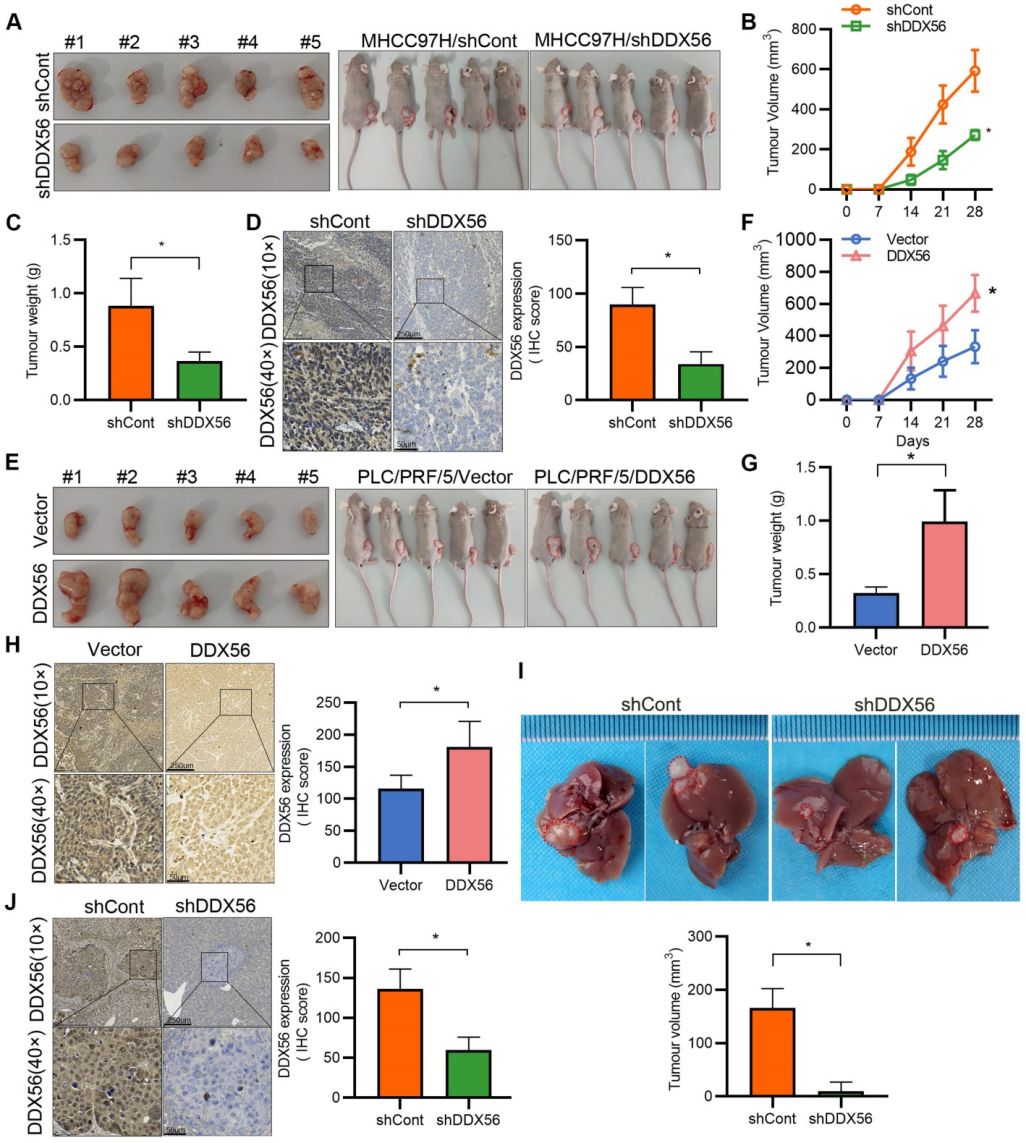
** Effect of DDX56 loss- or gain-of-function on the growth of HCC cells *in vivo.* (A-C)** Images of subcutaneous tumors in nude mice transplanted with LV-mediated shDDX56 and LV-control MHCC97H cells (A). Tumor volumes were measured every 7 d (B). The mice were sacrificed 28 d after cell injection and the tumors were removed and weighed (C). **(D)** Representative IHC images of DDX56 in paraffin-embedded section obtained from xenografts. Scale bars: 250 µm (top), 50 µm (bottom). **(E-H)** Effect of DDX56 gain-of-function in PLC/PRF/5 cells on subcutaneous tumor growth. Scale bars: 250 µm (top), 50 µm (bottom). **(I-J)** LV-mediated shDDX56 and LV-control MHCC97H cells were transplanted into the left lobe of the mouse liver. (I) Representative images showing livers with tumor lesions. Red circles indicate the primary liver tumor. (J) IHC staining showing DDX56 expression in mouse liver with tumor lesion. Scale bars: 250 µm (top), 50 µm (bottom).

To confirm the promotion of HCC by DDX56, stable DDX56-silenced cells and control cells were subcutaneously implanted near the thigh region of nude mice. Consistent with our *in vitro* observations, mice inoculated with DDX56-silenced cells potently suppressed tumor volume and tumor weight (Figure 3A-D). Conversely, DDX56 overexpression significantly increased xenograft tumor volume, tumor weight, and growth (Figure 3E-H). These results were further corroborated in an orthotopic HCC mouse model. DDX56 knockdown considerably inhibited the volume of HCC tumors in the orthotopic liver mouse model (Figures 3I-J). Overall, these results suggested that DDX56 may function as a tumor promoter in HCC.

### RNA expression profiling identifies MIST1 as a DDX56 downstream target

**Figure 4:**
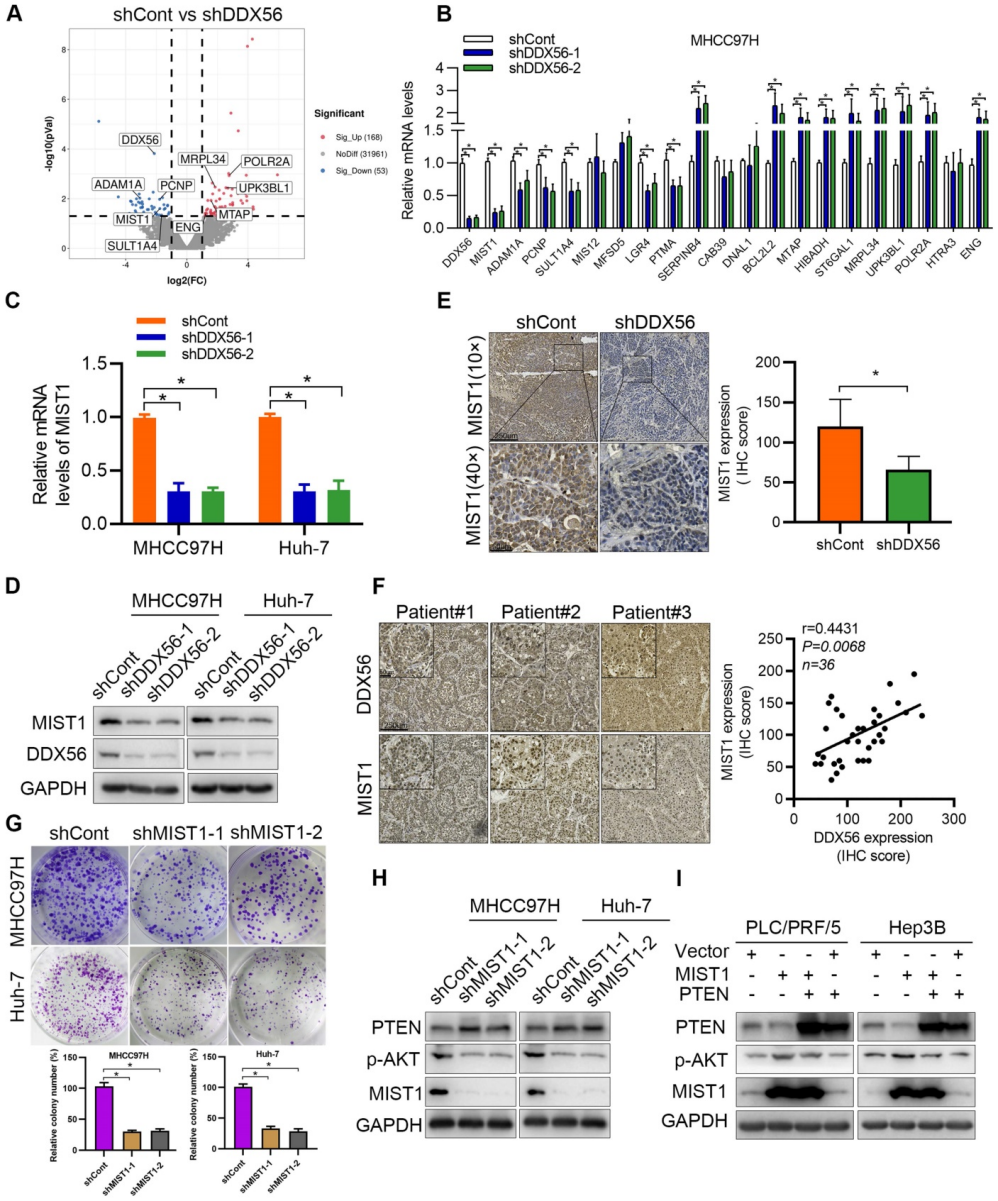
** RNA expression profiling identifies MIST1 as a DDX56-downstream target in the regulation of the PTEN-AKT axis. (A)** A volcano plot constructed using *P*-values and fold-change to illustrate the differential downstream target genes between DDX56-silenced MHCC97H cells and control cells (|fold change| ≥2, *P* < 0.05). Red dots represent significantly upregulated genes while blue dots represent significantly downregulated genes. **(B)** The top 20 differential downstream genes from RNA-seq results were validated by RT-PCR in MHCC97H cells. **(C-D)** The mRNA and protein levels of MIST1 in both MHCC97H and Huh-7 cells were detected using RT-PCR and western blotting, respectively. **(E)** IHC staining shows the expression level of MIST1 in the subcutaneous tumor. Scale bars: 250 µm (top), 50 µm (bottom). **(F)** Representative images of DDX56 and MIST1 immunohistochemical staining in human HCC tissues (left). Correlation analysis between DDX56 and MIST1 protein expression in HCC tissues (right). Scale bars: 250 µm (low magnification), 50 µm (high magnification). **(G)** Effect of MIST1 knockdown on MHCC97H and Huh-7 cell proliferation was determined by colony formation assay. **(H)** The indicated protein was determined using a western blot analysis of MHCC97H and Huh-7 cells infected with MIST1-silencing lentivirus (shMIST1) or lentivirus control (shCont). **(I)** Western blot analysis of the PTEN-AKT axis in MIST1-overexpressed HCC cells, showing that PTEN overexpression reversed the effect of MIST1 on the PTEN-AKT axis. Cells were transfected with 3 µg of the indicated plasmids for 72 h.

To explore the mechanism underlying DDX56-induced HCC proliferation, DDX56 knockdown cells as well as control cells were subjected to RNA sequencing (RNA-seq). Differential gene expression analysis revealed 168 upregulated and 53 downregulated genes (|fold-change| ≥2, *P* < 0.05); (Figure 4A). Gene Ontology (GO) functional analysis was performed to explore the biological processes and molecular functions of these differentially expressed genes (Figure S3A-C). The GO terms 'biological regulation', 'cell communication', 'cell proliferation', and 'growth' were enriched in biological processes, while 'nucleus' and 'protein-containing complex' were the top enriched terms in the cellular component, and 'protein binding' and 'enzyme regulator activity' were frequently enriched in molecular functions. Based on RNA-seq results, the top 20 differentially expressed genes with the most significant differences and the largest fold changes were selected for RT-PCR validation (Figure 4B and S3D). Interestingly, MIST1, also known as BHLHA15, was among the most drastically downregulated genes in both DDX56-silenced HCC cell lines. To investigate the clinical relevance of MIST1 expression in HCC, we first examined the mRNA levels of MIST1 using TCGA-LIHC. Compared to normal tissues, MIST1 mRNA levels were significantly upregulated in TCGA-LIHC tissues (Figure S3E). Next, we used RT-PCR and western blotting separately to determine the effect of DDX56 on MIST1 expression. Consistent with previous results, MIST1 expression in DDX56-silenced cells was significantly downregulated, whereas cells overexpressing DDX56 upregulated MIST1 expression (Figure 4C-D and S3F-G). IHC assays further revealed that MIST1 expression in DDX56-silenced xenograft tumors was reduced (Figure 4E). Moreover, DDX56 and MIST1 protein expression levels in primary HCC patient samples (n = 36) were positively correlated, suggesting that the DDX56/MIST1 axis played a clinically relevant role in HCC (Figure 4F). Importantly, consistent with the promoting effect exerted by DDX56 on HCC, MIST1 knockdown significantly inhibited the proliferation of HCC cells (Figure 4G and S4A-D). These data suggest that MIST1 plays a vital role in HCC cell proliferation.

MIST1 reportedly hijacks the PTEN-AKT signaling pathway to promote anoikic resistance in skin cancer 15. Therefore, we investigated whether MIST1 contributes to HCC development by regulating the PTEN-AKT pathway. Significant upregulation of PTEN and downregulation of AKT phosphorylation were observed in MIST1-silenced HCC cells (Figure 4H). Moreover, the results showed that PTEN overexpression had rescued the MIST1-mediated effect that promotes p-AKT expression (Figure 4I). Oroxin B, an agonist of PTEN, plays an important role in anti-tumor treatment 22, 23. Importantly, cells overexpressing MIST1 were treated with Oroxin B, and these cells abrogated the effect of MIST1-mediated PTEN downregulation and p-AKT upregulation (Figure S5A), indicating that MIST1-mediated HCC development relies on PTEN expression, at least partially. We next explored whether MIST1, which is a transcription factor, regulates PTEN via transcriptional regulation. A ChIP-PCR assay demonstrated that MIST1 did not bind to different regions of the PTEN promoter in any significant manner (Figure S5B), suggesting that the regulatory role played by MIST1 in the functioning of PTEN may not occur at the transcriptional level. Previous studies have shown that SNAI1 may be involved in the regulation of PTEN expression by MIST1 15, 16, 24. To test this assumption, we first examined the effect of MIST1 knockdown on SNAI1 expression. Our findings showed that MIST1 knockdown reduced the mRNA and protein levels of SNAI1 (Figure S5C-D). Moreover, SNAI1 overexpression markedly rescued MIST1 knockdown-induced upregulation of PTEN expression and downregulation of AKT phosphorylation (Figure S5E), suggesting that SNAI1 is involved in the MIST1-mediated PTEN/AKT signaling pathway. Furthermore, the ChIP assay revealed that MIST1 directly binds to the SNAI1 promoter and that DDX56 knockdown substantially reduces occupancy of the promoter of* SNAI1* by MIST1 (Figure S5F). Therefore, these results indicate that MIST1 inhibits the expression of PTEN by activating SNAI1 transcription.

**Figure 5:**
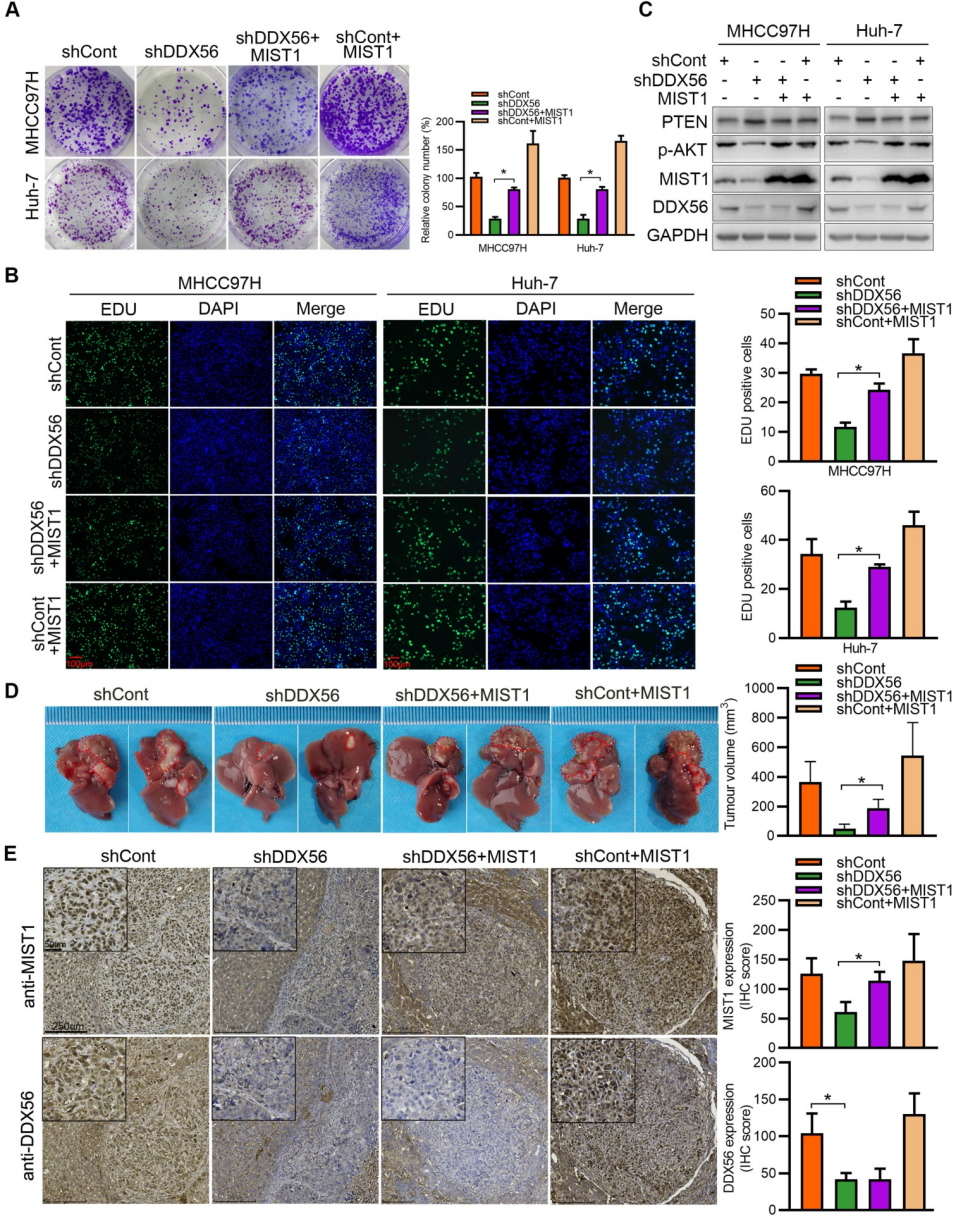
** DDX56 facilitates HCC growth in a MIST1-dependent manner. (A-B)** Colony formation (A) and EdU assays (B) revealed that ectopic MIST1 expression in DDX56-silenced cells partially reversed the inhibitory effect on HCC proliferation. Scale bars: 100 µm. **(C)** Western blot analysis of the PTEN-AKT axis in DDX56-silenced HCC cells showing that MIST1 overexpression reversed the effect of DDX56 on the PTEN-AKT axis. **(D-E)** DDX56 knockdown decreased liver tumor formation capacity of HCC cells in the orthotopic HCC implantation models, and the effect was reversed by simultaneously overexpressing MIST1. (D) Representative images showing mouse livers with tumor lesions. Red circles indicate the primary liver tumor. (E) Representative images of IHC staining of DDX56 and MIST1 in paraffin-embedded sections obtained from mouse orthotopic HCC models. Scale bars: 250 µm (low magnification), 50 µm (high magnification).

To determine whether DDX56 facilitates HCC development in a MIST1-dependent manner, we analyzed the role of DDX56 knockdown-mediated MIST1 suppression in HCC proliferation. Although DDX56 knockdown significantly inhibited HCC proliferation, suppression of DDX56 knockdown was partially abrogated by MIST1 overexpression *in vitro* (Figure 5A-B and S6A). Similarly, the results indicated that DDX56 knockdown also elevated the protein level of PTEN and repressed phosphorylation of AKT (Figure S6B), whereas the inhibitory effect mediated by DDX56 knockdown was restored by MIST1 overexpression (Figure 5C). Furthermore, this phenomenon was confirmed in a mouse orthotopic hepatoma nude model. Tumor volumes were significantly reduced following the implantation of stable DDX56 knockdown cells, compared to control cells, whereas MIST1 overexpression partially abrogated the suppressive effects exerted by DDX56 knockdown on tumor formation (Figures 5D-E). Collectively, these data suggested that DDX56-silencing suppresses tumorigenesis in an MIST1-dependent manner.

### DDX56 directly activates the transcription of MIST1 in HCC cells

**Figure 6:**
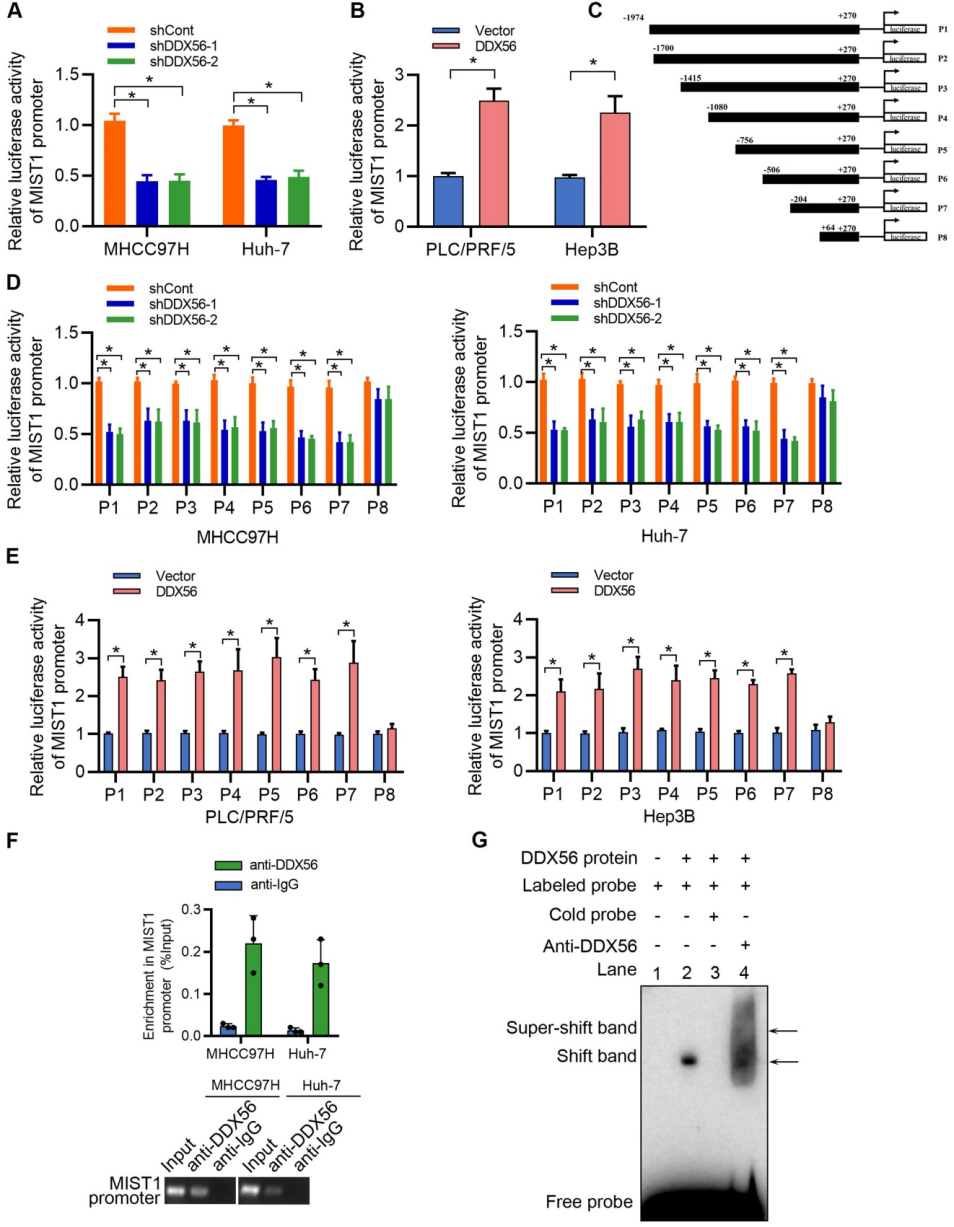
** DDX56 activates MIST1 transcription by directly binding to the promoter of MIST1. (A-B)** MHCC97H and Huh-7 cells were co-transfected with MIST1 promoter-luciferase constructs and DDX56 silencing (A) or DDX56 overexpression (B) plasmids. Firefly luciferase activity was detected and Renilla luciferase activity of pRL-TK was used for normalization. **(C)** Schematic of a series of luciferase reporter (P1-P8) constructs containing different fragments of the MIST1 promoter region. **(D-E)** Luciferase activity assay for P1-P8 fragments of the MIST1 promoter in DDX56-loss-of-function cells (D) or DDX56-gain-of-function cells (E). **(F)** Chromatin immunoprecipitation (ChIP) assay showing enrichment of DDX56 binding to the MIST1 promoter in MHCC97H and Huh-7 cells. DDX56 binding at the MIST1 promoter region is shown relative to input. IgG was used as a negative control (top). Agarose gel electrophoresis of PCR fragments after ChIP (bottom). **(G)** Electrophoretic mobility shift assay (EMSA) detecting the binding of recombinant DDX56 to the MIST1 promoter. Lane1: Only biotin-labelled probes are added; Lane2: Biotin-labelled probes and proteins are added; Lane3: Biotin-labelled probes, proteins, and competitive probes are added; Lane4: Biotin-labelled probes, proteins, and DDX56 antibody are added.

DDX56, a transcription factor, plays an important role in the transcriptional regulation of downstream targets. To investigate the mechanisms underlying the regulatory effect exerted by DDX56 on MIST1 expression, reporter luciferase activities were determined using a dual-luciferase reporter system. We found that silencing DDX56 inhibited the luciferase activity of the MIST1 promoter in HCC cells, whereas upregulating DDX56 enhanced it (Figure 6A-B). Moreover, luciferase reporters containing eight fragments of MIST1 were constructed as follows: P1 (-1974 to +270); P2 (-1700 to +270); P3 (-1415 to +270); P4 (-1080 to +270); P5 (-756 to +270); P6 (-506 to +270); P7 (-204 to +270); and P8 (+64 to +270); (Figure 6C). Dual-luciferase reporter assays showed that the luciferase activity of fragments P1-P7 was significantly downregulated in DDX56-silenced MHCC97H and Huh-7 cells, but upregulated in DDX56-overexpressing PLC/PRF/5 and Hep3B cells (Figure 6D-E). However, no significant change in the luciferase activity of fragment P8 was observed, suggesting that the fragment between -204 to + 64 bp was essential for driving the transcription of MIST1 (Figure 6D-E). Furthermore, the ChIP-PCR assay revealed that DDX56 binds to the MIST1 promoter region in HCC cells (Figure 6F). To further confirm these results, recombinant human DDX56 protein and 6 biotin-labelled DNA probes covering MIST1 promoter sequences (-204 to +64 bp) were utilized for the EMSA assay. Recombinant DDX56 bound exclusively with the -160 to -117bp region of the MIST1 promoter to form a band exhibiting electrophoretic mobility (Figure S7A). Furthermore, the binding of the biotinylated probe was inhibited in the presence of a competitive, unlabeled DNA probe. Meanwhile, the addition of the DDX56 antibody to the reaction resulted in the formation of a super-shifted band, confirming the specificity of the DDX56-MIST1 complex (Figure 6G). Considered together, these findings suggested that DDX56 activates MIST1 transcription by directly binding to the MIST1 promoter.

### DDX56 interacts with MECOM to promote MIST1 expression

**Figure 7:**
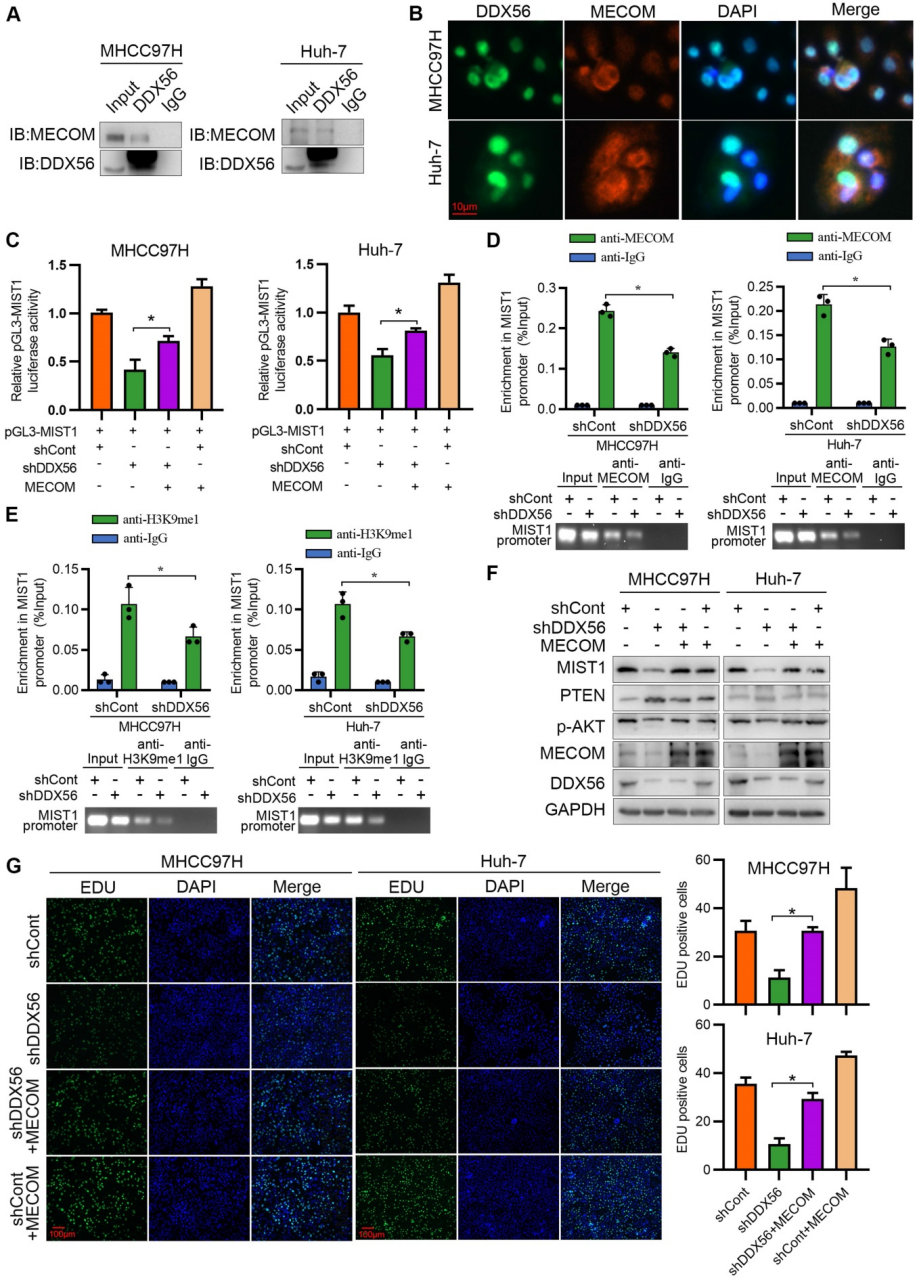
** DDX56 promotes MIST1 transcription by recruiting the epigenetic regulator MECOM. (A)** Co-immunoprecipitation experiment showed the interaction between DDX56 and MECOM in MHCC97H and Huh-7 cells. **(B)** Immunofluorescence analysis shows the co-localization of DDX56 (green) and MECOM (red). **(C)** DDX56 rescues MECOM to regulate MIST1 promoter-driven luciferase activity. DDX56-knockdown HCC cells were co-transfected with MIST1 promoter luciferase constructs and MECOM overexpression plasmid. Cell lysates were measured for firefly and Renilla (RL-TK) activity. **(D-E)** ChIP-qPCR assay with anti-MECOM or anti-H3K9me1 was performed in MHCC97H and Huh-7 cells. DDX56 loss-of-function reduced MECOM (D) or H3K9me1 (E) occupancy at the promoter of MIST1 gene (top). Agarose gel electrophoresis of PCR fragments after ChIP (bottom). **(F)** Western blot analysis of MIST1/PTEN/AKT axis in DDX56-silenced HCC cells, and MECOM overexpression reversed the effect of DDX56 on the MIST1/PTEN/AKT axis. **(G)** EdU assays revealed that ectopic MECOM expression in DDX56-silenced cells partially reversed the DDX56 knockdown-mediated inhibitory effect on HCC proliferation. Scale bars: 100 µm.

To better understand the mechanisms underlying the regulation of HCC proliferation by DDX56 and MIST1, we analyzed the binding between proteins and DDX56 via mass spectrometry (MS). Transcription is tightly controlled by epigenetic regulation 25. Therefore, we focused on proteins that usually function as epigenetic regulators (Table S7). MECOM, a member of the transcriptional regulator family, is of particular interest as it can contribute to the tumorigenesis of HCC 26, 27, and displays histone methyltransferase activity in the monomethylation of Lys9 of histone H3 (H3K9me1) *in vitro* 28, 29. Reciprocal co-immunoprecipitation experiments showed that MECOM specifically binds to DDX56 (Figure 7A), while immunofluorescence experiments demonstrated that DDX56 and MECOM were colocalized in HCC cells (Figure 7B). Moreover, a dual-luciferase reporter assay indicated that MECOM overexpression partially reversed a DDX56 knockdown-mediated reduction in reporter activity, suggesting that DDX56 knockdown-mediated suppression of MIST1 transcription was partially dependent on MECOM (Figure 7C). To confirm the binding of MECOM to the MIST1 promoter, a ChIP assay was performed. Silencing DDX56 inhibited the binding between MECOM and the MIST1 promoter (Figure 7D). By contrast, DDX56 overexpression was sufficient to enhance the binding of MECOM to the MIST1 promoter (Figure S8A). MECOM has been identified as an H3K9me1-specific lysine methyltransferase (KMT) which enables histone methylation and thereby transcriptional activation 28, 29. To further elucidate the molecular mechanism underlying the binding between MECOM and the MIST1 promoter, ChIP assays were performed to detect enrichment of H3K9me1 on the MIST1 promoter. Enrichment of H3K9me1 on the MIST1 promoter was reduced in DDX56-silenced HCC cells, whereas H3K9me1 modification of the MIST1 promoter was enhanced in HCC cells overexpressing DDX56 (Figure 7E and S8B). These results demonstrated that the recruitment of MECOM to the MIST1 promoter by DDX56 enhances MIST1 transcription via H3K9 monomethylation. To determine whether MECOM is involved in the DDX56-mediated signaling pathway, we first knocked down MECOM and found that MIST1 expression was significantly reduced (Figure S9A). Furthermore, our results showed that overexpression of MECOM in DDX56-silenced cells rescued the suppressive effect of MIST1 expression and the PTEN-AKT pathway (Figure 7F). EdU assays revealed that MECOM overexpression significantly mitigated the inhibitory effect of DDX56 knockdown on HCC cell proliferation (Figure 7G). Correlation analyses of data obtained from the TCGA-LIHC database (r = 0.3467; *P* < 0.0001) revealed that MECOM expression was positively correlated with MIST1 expression (Figure S9B). Western blot analyses of 24 pairs of HCCs collected in our laboratory consistently demonstrated that MECOM and MIST1 expression were positively correlated (r = 0.5670; *P* = 0.0039); (Figure S9C), indicating the clinical relevancy of MECOM/MIST1 in HCC specimens. Considered together, these results indicated that DDX56 enhances MIST1 transcription by recruiting MECOM to mono-methylate H3K9 on the MIST1 promoter, which subsequently activates the PTEN-AKT pathway to promote HCC proliferation.

### ZEB1 directly activates the transcription of DDX56 in HCC cells

**Figure 8:**
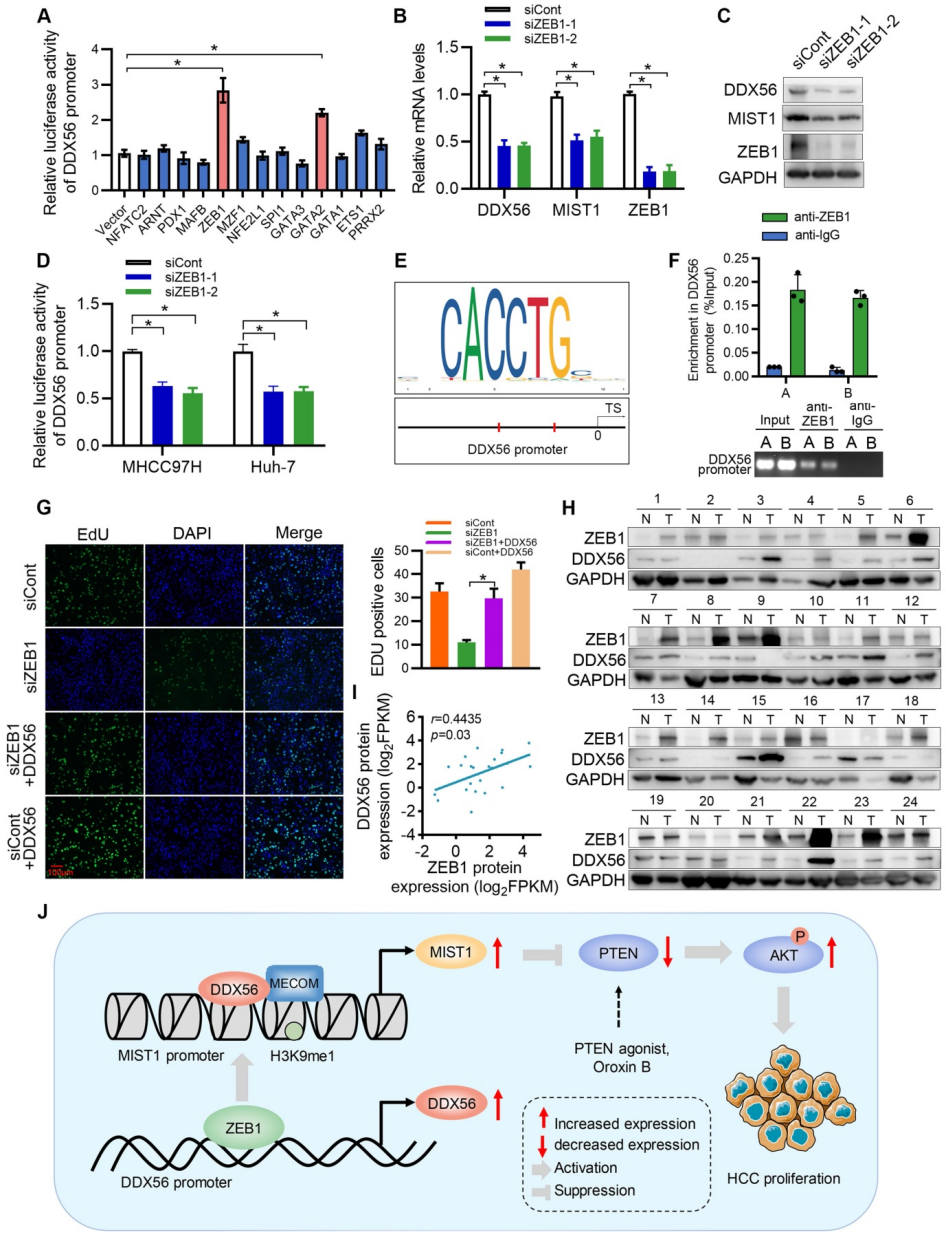
** ZEB1 binds to the DDX56 promoter and facilitates transcription of DDX56. (A)** HEK293T cells were co-transfected with the DDX56-luciferase construct together with the indicated plasmids for 48 h. **(B-C)** mRNA (B) and protein (C) levels of indicated genes were detected using RT-PCR and western blotting, respectively. **(D)** MHCC97H and Huh-7 cells were co-transfected with DDX56 promoter luciferase constructs and ZEB1-silencing molecules. **(E)** Computational prediction of ZEB1 binding sites on DDX56 promoter region (-2000 to +1) by JASPAR and MEM-suite matrix model. **(F)** ChIP-PCR assay was performed using anti-ZEB1 to confirm the two predicted ZEB1-binding sites on the DDX56 promoter. **(G)** EdU assay showed that ectopic DDX56 expression in ZEB1-silenced cells partially reversed the ZEB1 loss-of-function-mediated inhibitory effect on HCC proliferation. Scale bars: 100 µm. **(H-I)** Protein levels of ZEB1 and DDX56 in HCC tissues and corresponding adjacent normal tissues (n = 24) were examined using western blot analysis (H), followed by an analysis of their correlation (r = 0.4435, *P* = 0.03) (I). Protein band intensity was quantitated using Image J software. **(J)** A schematic model showing the ZEB1-DDX56-MIST1 axis regulating HCC proliferation. ZEB1 directly binds to the promoter region of DDX56 and promotes its transcription. Upregulated DDX56 enhances MIST1 transcription by recruiting MECOM and mono-methylating H3K9 at the MIST1 promoter, which subsequently reduces PTEN protein and facilitates AKT phosphorylation, to promote HCC proliferation. Importantly, the PTEN agonist, Oroxin B (OB), blocked the DDX56-mediated PTEN-AKT signaling pathway, suggesting that treating of HCC patients with OB may result in a beneficial therapeutic intervention.

Although our study established DDX56 dysregulation as being important for HCC proliferation, the mechanism(s) underlying DDX56 upregulation in HCCs remains poorly understood. To investigate this issue, we used the JASPAR database to determine possible upstream regulators of the DDX56 promoter and identified 13 candidate genes. Results of the analysis indicated that both ZEB1 and GATA2 enhanced luciferase reporter activity by more than 2-fold (Figure 8A). Furthermore, we targeted two candidate genes for transfection with siRNA and found that siRNA-based targeting of ZEB1, but not of GATA2, significantly reduced the expression levels of DDX56 and MIST1 (Figs.8B-C and S10A-B). Moreover, silencing ZEB1 inhibited luciferase reporter activity of DDX56 in HCC cells (Figure 8D). Interestingly, according to the JASPAR and MEME Suite databases, two ZEB1 binding sites were predicted in the promoter region of DDX56 with a high relative score of > 0.90 (Figure 8E). ChIP assays revealed that ZEB1 directly binds to the DDX56 promoter (Figure 8F). To determine the effect of ZEB1 on HCC proliferation, we assessed the mRNA levels of ZEB1 in 371 HCC patients using TCGA-LIHC (Figure S10C). Our results showed that ZEB1 expression in HCC was upregulated and that high levels of ZEB1 were significantly associated with poor prognoses for HCC (Figure S10D). Importantly, the EdU assay showed that ZEB1 downregulation significantly inhibited HCC proliferation, whereas DDX56 overexpression partly rescued the inhibitory effects of ZEB1 knockdown, suggesting that ZEB1 may play an important role in regulating DDX56 expression (Figure 8G). The physiological relevance of these findings was further substantiated by the finding that the mRNA level of ZEB1 was positively correlated with the mRNA level of DDX56 in HCC tissues from the TCGA-LIHC database (Figure S10E). Furthermore, western blot analyses also revealed a positive correlation between the protein expression levels of ZEB1 and DDX56 in 24 pairs of HCCs collected in our laboratory (r = 0.4435; *P* = 0.03); (Figure 8H-I). Considered together, these data indicated that ZEB1 may directly bind to DDX56 and activate DDX56 transcription to promote HCC proliferation.

## Discussion

RNA-binding proteins (RBPs) have been shown to play a key role in carcinogenesis. Several RBPs, which are highly expressed in solid tumors, such as colorectal carcinoma 30, breast cancer 31, lung adenocarcinoma 32 and liver cancer 33, have been identified as drivers of carcinogenesis. These proteins are highly stable, conserved, and tightly regulated in cancer cells. Dysregulation of RBPs may cause large-scale changes in global gene expression levels and lead to significant transcriptomic imbalances. Therefore, in order to increase the effectiveness and specificity of current therapies, it is necessary to discover new HCC therapeutic targets by elucidating biological mechanisms underlying aberrant RBP expression.

To identify RBPs that are responsible for HCC development, we used gene expression profiling analysis to compare HCC tumor tissues with their paired non-tumoral tissues. Further screening of RBPs found to be highly expressed in TCGA-LIHC revealed that they were mainly associated with high tumor grades as well as worse survival rates. In the present study, we identified one of the top gene signatures associated with HCC development. This gene encodes DDX56, an RNA helicase protein, which is a vital promoter of HCC proliferation and an independent prognostic marker, and thus we selected it for further functional investigation. DDX56 plays an essential role in the progression of multiple cancers, including squamous cell lung carcinoma 8, glioblastoma 9, osteosarcoma 10, and colorectal cancer 11. Wu et al. 8 showed that DDX56 exerts its oncogenic effects via miR-378a-3p-mediated post-transcriptional regulation of Wnt signaling genes, thereby promoting the recurrence of early squamous cell lung carcinoma (SqCLC). Additionally, Kouyama et al. reported that high expression levels of *DDX56*, which mediates the alternative splicing of WWE1, may be useful as a prognostic biomarker of colorectal cancer (CRC) 11. However, the functioning of DDX56 in HCC tumorigenesis has not yet been elucidated. The results of this study indicate that elevated expression levels of DDX56 in HCC tissues and cells are significantly associated with tumor grades and poor prognoses for HCC patients. Notably, *in vitro* HCC proliferation was promoted by DDX56 upregulation and inhibited by DDX56 knockdown. These observations were validated *in vivo* using mouse orthotopic liver xenograft tumor models and subcutaneous xenograft tumor models. The above result suggests that DDX56 plays a vital role in HCC, although the exact mechanism via which it does so may require further investigation.

MIST1 is a bHLH transcription factor that is essential for the development of secretory cells 34. Lee et al. reported that MIST1 overexpression leads to the regulation of SNAI1expression levels, which in turn hijacks the PTEN-AKT signaling pathway to promote the anoikic resistance capacity of melanoma cells 15. MIST1 promoted inflammation in a colitis model via K+-ATPase NLRP3 inflammasomes by regulating the expression of SNAI1 24. Moreover, Li et al. showed that MIST1 reduced the tumorigenicity of pancreatic cancer cells and reversed EMT, partly via its effect on the SNAI1/E-cadherin signaling pathway 16. These findings suggest that MIST1 plays an important role in cancer development. However, definitive evidence supporting the precise function of MIST1 in HCC is currently lacking. In the present study, we demonstrated that DDX56 knockdown significantly reduced the mRNA and protein levels of MIST1. The inhibitory effect of DDX56 knockdown on HCC proliferation was partially rescued by the exogenous expression of MIST1 *in vitro* and *in vivo*, suggesting that DDX56 may promote HCC proliferation by orchestrating MIST1 expression. Furthermore, we found that high MIST1 expression regulates the PTEN-AKT signaling pathway by activating SNAI1 transcription which in turn promotes HCC proliferation. Importantly, the PTEN agonist, Oroxin B (OB), blocks the DDX56-mediated PTEN-AKT signaling pathway. Given that OB is a flavonoid isolated from traditional Chinese herbal medicine, *Oroxylum indicum* (L.) Vent, which displays obvious inhibitory effects and induces early apoptosis on liver cancer cells, by upregulating PTEN 35, the above results suggest that treating HCC patients with OB may result in a beneficial therapeutic intervention.

DDX56, which is a component of 65S pre-ribosomal particles, regulates diverse RNA metabolism processes including transcription 36. Luciferase assays revealed that the MIST1 promoter region, (-204 to +64bp), was essential for DDX56-induced HCC proliferation. This observation was further substantiated by ChIP and EMSA assays, indicating that DDX56 may activate MIST1 transcription by directly binding to the promoter of MIST1. The Prdm family protein, MECOM, was identified as an H3K9me1-specific KMT 29, which was consistent with our results. Our results indicated that DDX56 enhances MIST1 transcription by recruiting MECOM to retain H3K9me1 at the MIST1 promoter, consequently promoting HCC proliferation. However, little is known regarding the causes of deregulated DDX56 expression in HCC.

Zinc finger E-box-binding homeobox 1 (ZEB1) is a transcription factor that reportedly promotes cancer proliferation in lung adenocarcinoma 37, HCC 38 and pancreatic cancer 39. Previous studies have shown that ZEB1 enhances HCC tumorigenesis by activating 6-phosphofructokinase, muscle type (PFKM) transcription 20. Our results demonstrate that ZEB1 directly binds to the promoter of DDX56 and activates DDX56 transcription, which provides an alternative and possibly complementary mechanism by which ZEB1 regulates HCC proliferation.

## Conclusions

In summary, our findings reveal that upregulated DDX56 is significantly related to poor prognoses for HCC patients, and that DDX56 silencing inhibits HCC proliferation *in vitro* as well as *in vivo*. Mechanistically, DDX56 interacts with MECOM and promotes HCC growth by mono-methylating H3K9 on the MIST1 promoter, leading to enhanced MIST1 transcription. Upregulated MIST1 consequently regulates the PTEN-AKT signaling pathway by activating SNAI1 transcription to promote HCC proliferation. Importantly, the PTEN agonist, Oroxin B (OB), blocked the DDX56-mediated PTEN-AKT signaling pathway, suggesting that treating HCC patients with a PTEN agonist, such as OB, may result in a beneficial therapeutic intervention. Furthermore, we demonstrated that DDX56, which is transcriptionally activated by ZEB1, promotes HCC proliferation by directly binding to the DDX56 promoter (Figure 8J). In summation, we were able to elucidate the role played by the ZEB1-DDX56-MIST1 axis in HCC, and our findings suggest that targeting the components of this axis may present a beneficial therapeutic approach for the treatment of HCC.

## Supplementary Material

Supplementary figures and tables.

## Abbreviations

BHLHA15: basic helix-loop-helix family member a 15
CCK8: cell counting kit-8
ChIP: chromatin immunoprecipitation
EMSA: electrophoretic mobility shift assay
DDX56: dead-box 56
EdU: 5-Ethynyl-2'-deoxyuridine
H3K9me1: histone h3 lysine 9 mono-methylation
HCC: hepatocellular carcinoma
L02: immortalized normal liver cells
OB: oroxin B
PTEN: phosphatase and tensin homolog deleted on chromosome ten
PVDF: polyvinylidene difluoride
RBP: rna binding protein
RNA-seq: rna sequencing
MECOM: mds1 and evi1 complex locus
MIST1: muscle intestine stomach expression 1
MS: mass spectrometry
ZEB1: zinc finger e-box binding homeobox 1
