# Supplementary material for: DDX56 transcriptionally activates MIST1 to facilitate tumorigenesis of HCC through PTEN-AKT signaling
Source: Theranostics. 2022 Aug 15;12(14):6069–87. doi: 10.7150/thno.72471 (PMC9475456; doi:10.7150/thno.72471)
Supplement: Supplementary file 1 — Supplementary figures and tables. [file thnov12p6069s1.pdf]

DDX56 transcriptionally activates MIST1 to facilitate tumorigenesis of HCC through PTEN-AKT signalling

Supplementary Figure

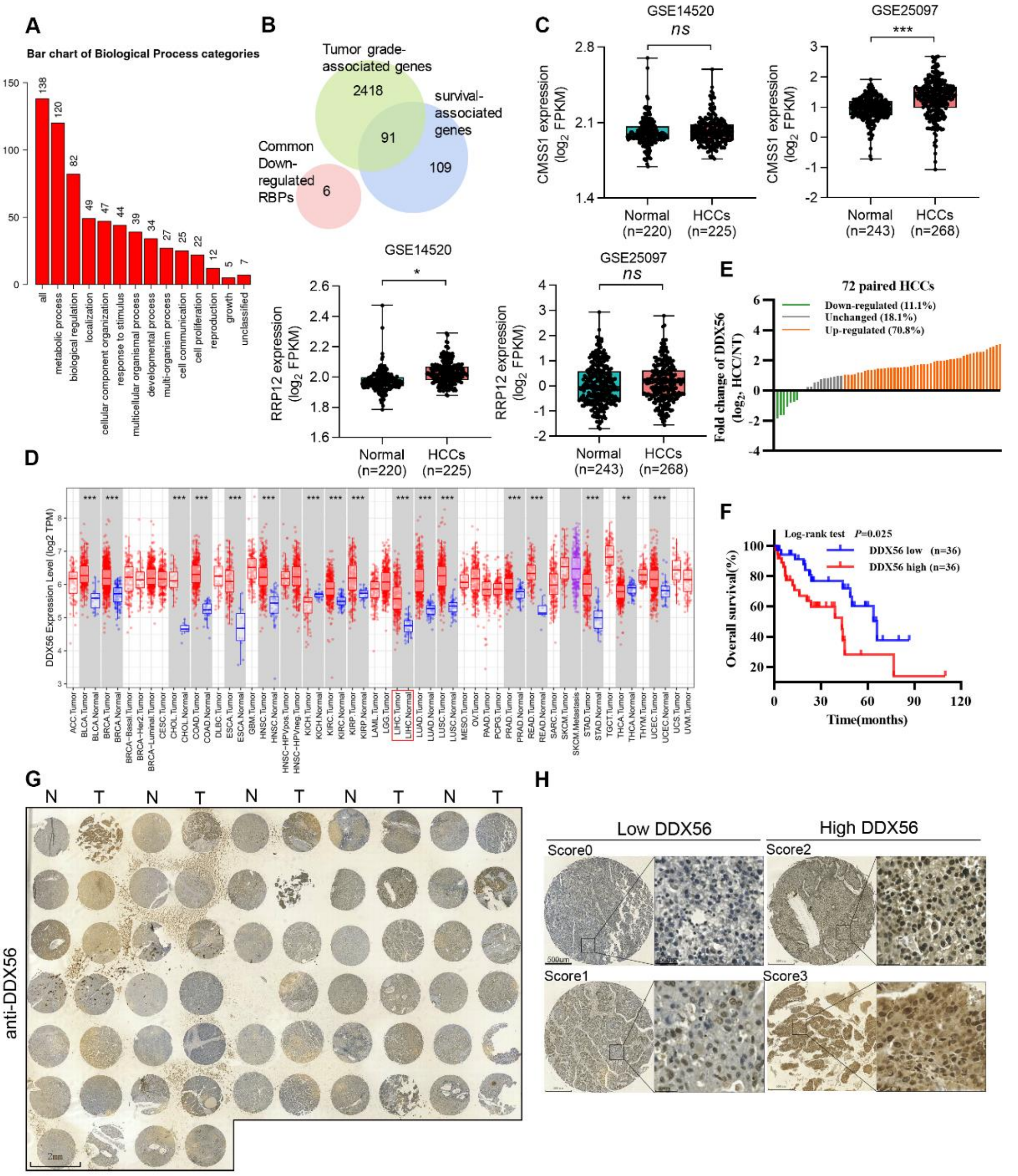

Figure S1 Detection of the significant upregulation of DDX56 in human HCC patients. (A) Gene Ontology (GO) analysis of differentially expressed RBP genes. (B) Venn diagram analyses of 6 downregulated RPBs, the top 200 survival-associated genes, and tumor grade-associated genes (P < 0.05). (C) Relative mRNA levels of CMSS1 or RRP12 in both GSE14520 and GSE25097 (* = P < 0.05; *** = P < 0.0001). (D) The level of DDX56 expression in all tumor tissues and adjacent non-tumor tissues from the TIMER database (* = P < 0.05 ** = P < 0.01; *** = P < 0. 001). (E) The mRNA level of DDX56 was detected in 72 freshly prepared HCC samples using RT-PCR. (F) Kaplan-Meier curves describe the correlation between DDX56 mRNA level and the overall survival of HCC patients. (G) Representative images of immunohistochemical staining of DDX56 from the HCC tissue array. Scale bars: 2 mm. (H) HCC samples in tissue array were immunostained with anti-DDX56 antibody. Representative images are shown. Scale bars: 500 µm (low magnification), 50 µm (high magnification).

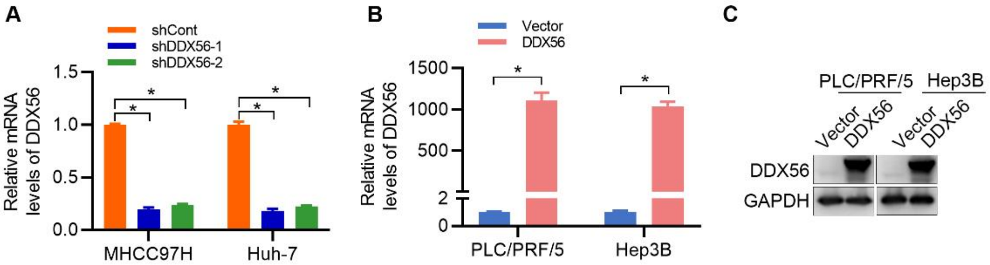
A
shCont
shDDX56-1
shDDX56-2
Relative mRNA levels of DDX56
1.5
1.0
0.5
0.0
MHCC97H
Huh-7
B
Vector
DDX56
Relative mRNA levels of DDX56
1500
1000
500
2
0
PLC/PRF/5
Hep3B
C
PLC/PRF/5
Hep3B
Vector
DDX56
Vector
DDX56
DDX56
GAPDH

Figure S2 Effect of DDX56 silencing or overexpression in HCC cells. (A–B) The mRNA level of DDX56 in DDX56-silencing or overexpressed HCC cells was detected using RT-PCR. (C) The effect of overexpressing DDX56 in DDX56 overexpressed HCC cells was detected via western blotting.

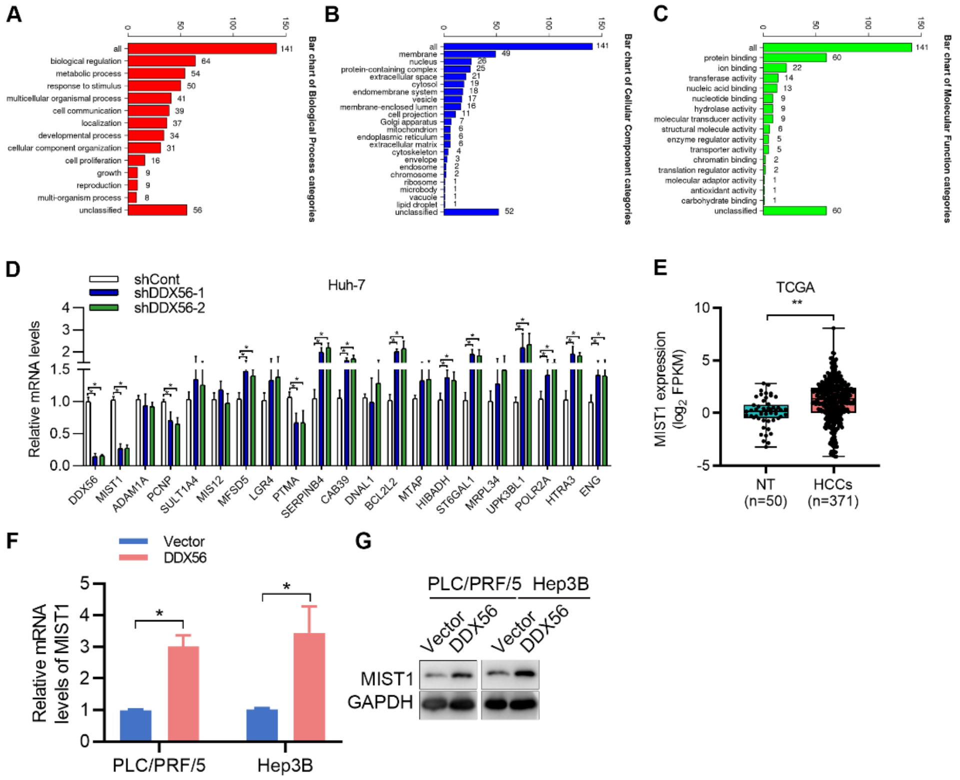
A
Bar chart of Biological Process categories
all 141
biological regulation 64
metabolic process 54
response to stimulus 50
multicellular organismal process 41
cell communication 39
localization 37
developmental process 34
cellular component organization 31
cell proliferation 16
growth 9
reproduction 9
multi-organism process 8
unclassified 56
B
Bar chart of Cellular Component categories
all 141
membrane 49
nucleus 26
protein-containing complex 25
extracellular space 21
cytosol 19
endomembrane system 18
vesicle 17
membrane-enclosed lumen 16
cell projection 11
Golgi apparatus 7
mitochondrion 6
endoplasmic reticulum 6
extracellular matrix 6
cytoskeleton 4
envelope 3
endosome 2
chromosome 2
ribosome 1
microbody 1
vacuole 1
lipid droplet 1
unclassified 52
C
Bar chart of Molecular Function categories
all 141
protein binding 60
ion binding 22
transferase activity 14
nucleic acid binding 13
nucleotide binding 9
hydrolase activity 9
molecular transducer activity 9
structural molecule activity 6
enzyme regulator activity 5
transporter activity 5
chromatin binding 2
translation regulator activity 2
molecular adaptor activity 1
antioxidant activity 1
carbohydrate binding 1
unclassified 60
D
Huh-7
shCont
shDDX56-1
shDDX56-2
Relative mRNA levels
DDX56
MIST1
ADAM1A
PCNP
SULT1A4
MIS12
MFSD5
LGR4
PTMA
SERPINB4
CAB39
DNAL1
BCL2L2
MTAP
HIBADH
ST6GAL1
MRPL34
UPK3BL1
POLR2A
HTRA3
ENG
E
TCGA
MIST1 expression (log2 FPKM)
NT (n=50)
HCCs (n=371)
F
Vector
DDX56
Relative mRNA levels of MIST1
PLC/PRF/5
Hep3B
G
PLC/PRF/5
Hep3B
Vector
DDX56
MIST1
GAPDH

Figure S3 Effect of DDX56 on MIST1 expression in HCC cells. (A–C) Gene ontology (GO) analysis of the differentially expressed genes, including biological process (A), cellular component (B), and molecular function (C) and (D). The top 20 differentially expressed downstream genes from the RNA-seq results were identified using RT-PCR of Huh-7 cells. (E) The expression of MIST1 was analyzed in 371 HCC tissues and 50 normal tissues from the TCGA-LIHC database. (F–G) The effect of DDX56 overexpression on MIST1 expression was determined using RT-PCR (F) and western blotting (G), respectively.

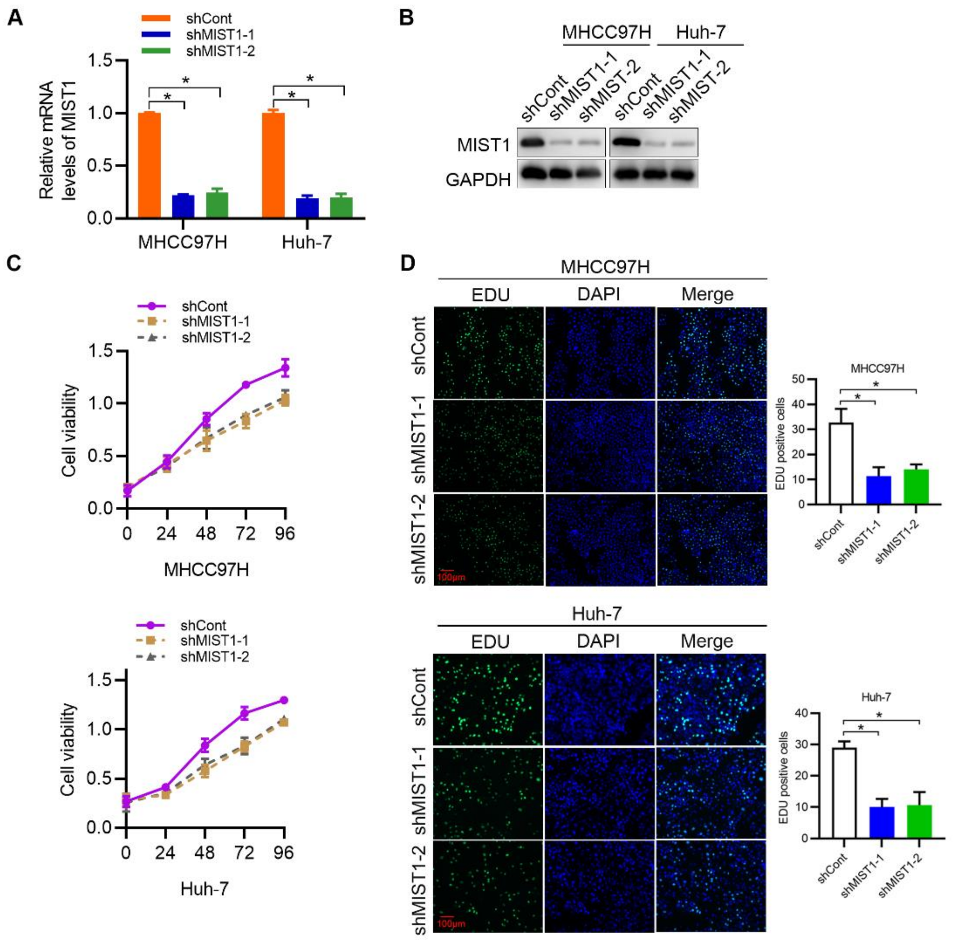

A
shCont
shMIST1-1
shMIST1-2
Relative mRNA levels of MIST1
MHCC97H
Huh-7
B
MHCC97H
Huh-7
shCont
shMIST1-1
shMIST-2
MIST1
GAPDH
C
Cell viability
MHCC97H
Huh-7
D
EDU
DAPI
Merge
EDU positive cells
100μm

Figure S4 Effect of MIST1 knockdown on HCC proliferation. (A–B) The effect of silencing MIST1 was detected using RT-PCR (A) and western blotting (B). (C) The effect of MIST1 knockdown on HCC cell viability was determined using CCK8. (D) The effect of MIST1 knockdown on DNA synthesis was assessed using EdU immunofluorescence staining. The graph on the right depicts the percentage of Edu-positive nuclei. Scale bars: 100 μm.

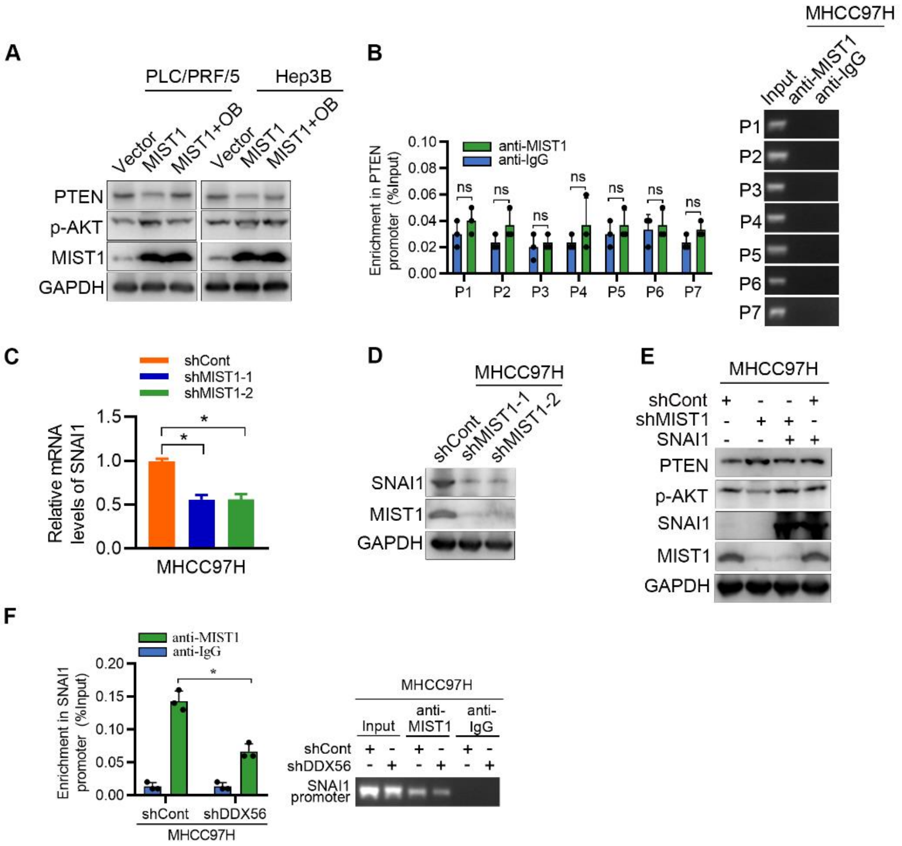
A
PLC/PRF/5
Hep3B
Vector
MIST1
MIST1+OB
PTEN
p-AKT
MIST1
GAPDH
B
MHCC97H
Input
anti-MIST1
anti-IgG
Enrichment in PTEN promoter (%Input)
anti-MIST1
anti-IgG
ns
P1
P2
P3
P4
P5
P6
P7
C
shCont
shMIST1-1
shMIST1-2
Relative mRNA levels of SNAI1
MHCC97H
D
MHCC97H
shCont
shMIST1-1
shMIST1-2
SNAI1
MIST1
GAPDH
E
MHCC97H
shCont
shMIST1
SNAI1
PTEN
p-AKT
SNAI1
MIST1
GAPDH
F
anti-MIST1
anti-IgG
Enrichment in SNAI1 promoter (%Input)
shCont
shDDX56
MHCC97H
MHCC97H
Input
anti-MIST1
anti-IgG
shCont
shDDX56
SNAI1 promoter

Figure S5 MIST1 inhibits PTEN expression by activating SNAI1 transcription. (A) The regulatory role of MIST1 in the PTEN-AKT axis was tested via treatment with 2 μM of Oroxin B (OB), a PTEN agonist (SMB00340, Sigma, USA), for 12 h. The indicated proteins in PLC/PRF/5 and Hep3B cells were detected using western blotting. (B) (Left) ChIP assay showing enrichment of MIST1 binding to different regions of the PTEN promoter in MHCC97H. MIST1 binding at the PTEN promoter region is shown relative to input. IgG was used as a negative control. (Right) Agarose gel electrophoresis of PCR fragments after ChIP. (C–D) The effect of MIST1 knockdown on the mRNA (C) and protein (D) levels of SNAI1 in MHCC97H. (E) Western blot analysis revealed SNAI1 overexpression reversed the effect of MIST1 knockdown on the SNAI1/PTEN/AKT axis. This indicated that the proteins were accurately determined by western blot analysis. (F) ChIP-PCR assay with anti-MIST1 was performed in MHCC97H cells. DDX56 knockdown reduced MIST1 occupancy at the promoter region of the SNAI1 gene (left). Agarose gel electrophoresis of PCR fragments after ChIP (right).

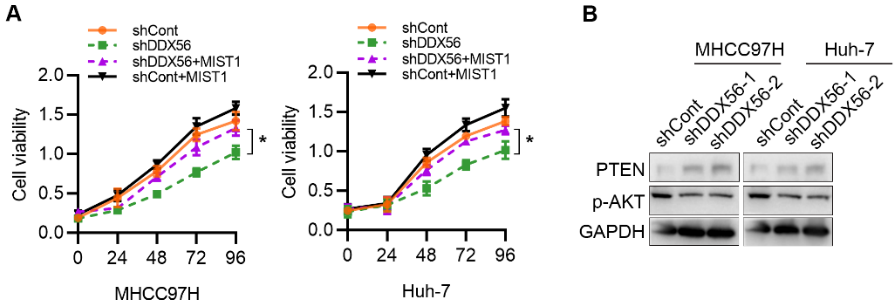
A
shCont
shDDX56
shDDX56+MIST1
shCont+MIST1
Cell viability
2.0
1.5
1.0
0.5
0.0
0
24
48
72
96
*
MHCC97H
Huh-7
B
MHCC97H
Huh-7
shCont
shDDX56-1
shDDX56-2
PTEN
p-AKT
GAPDH

Figure S6 DDX56 regulation of HCC proliferation is partly dependent on MIST1 expression. (A) CCK8 assays identified that the ectopic MIST1 expression in DDX56-silenced cells partially reversed the DDX56 knockdown-mediated inhibitory effect on HCC cell viability. (B) The indicated proteins in DDX56-silencing HCC cells were detected using western blot analysis.

A

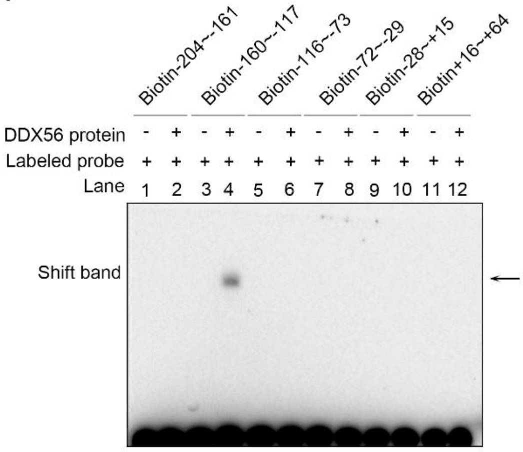
Biotin-204~-161
Biotin-160~-117
Biotin-116~-73
Biotin-72~-29
Biotin-28~+15
Biotin+16~+64
DDX56 protein - + - + - + - + - + - +
Labeled probe + + + + + + + + + + + +
Lane 1 2 3 4 5 6 7 8 9 10 11 12
Shift band

Figure S7 DDX56 directly binds to the promoter of MIST1. (A) EMSA detected the binding of recombinant DDX56 to different regions of the MIST1 promoter.

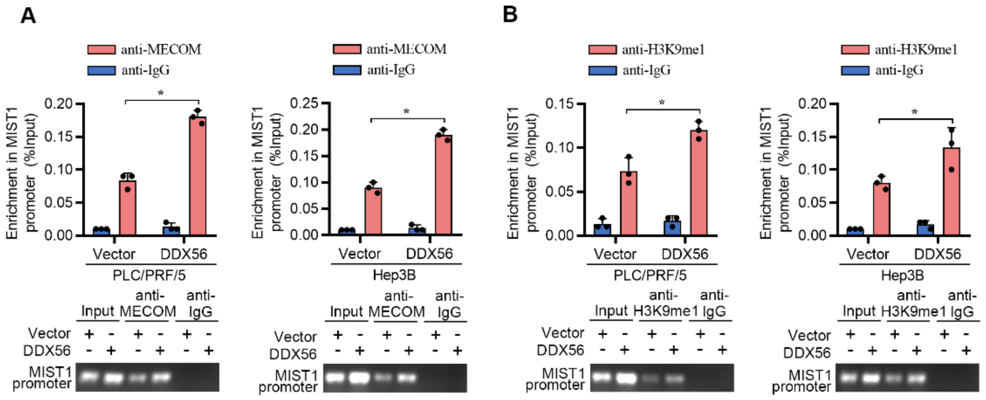

Figure S8 Effect of DDX56 overexpression on MECOM or H3K9me1 binding to the MIST1 promoter in HCC cells. (A–B) A ChIP-qPCR assay with anti-MECOM or anti-H3K9me1 was performed in PLC/PRF/5 and Hep3B cells. DDX56 gain-of-function promoted MECOM (A) or H3K9me1 (B) occupancy at the promoter region of MIST1 (top). Agarose gel electrophoresis of PCR fragments after ChIP (bottom).

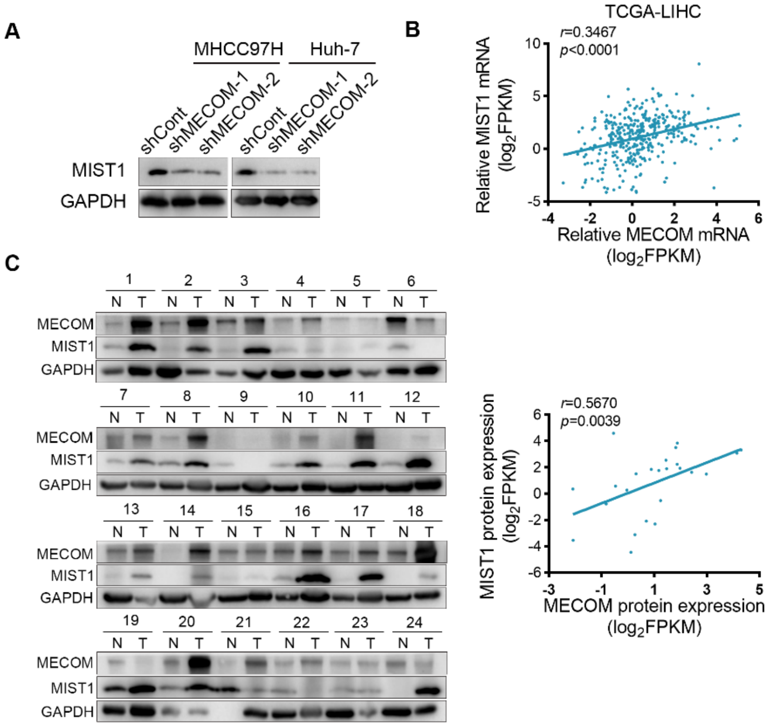

A
MHCC97H
Huh-7
shCont
shMECOM-1
shMECOM-2
MIST1
GAPDH
B
TCGA-LIHC
r=0.3467
p<0.0001
Relative MIST1 mRNA (log2FPKM)
Relative MECOM mRNA (log2FPKM)
C
MECOM
MIST1
GAPDH
N
T
r=0.5670
p=0.0039
MIST1 protein expression (log2FPKM)
MECOM protein expression (log2FPKM)

Figure S9 Effect of MECOM knockdown on MIST1 expression in HCC cells. (A) The expression of MIST1 in MECOM knockdown cells was detected using western blot analysis. (B) Correlation between MECOM and MIST1 mRNA levels in the TCGA-LIHC database (r = 0.3467; $P < 0.0001$). (C) The protein levels of MECOM and MIST1 in HCC tissues and the corresponding adjacent normal tissues (n = 24) were examined using western blot analysis, followed by an analysis of their correlation (r = 0.5670, $P = 0.0039$). Protein band intensity was quantified using Image J software.

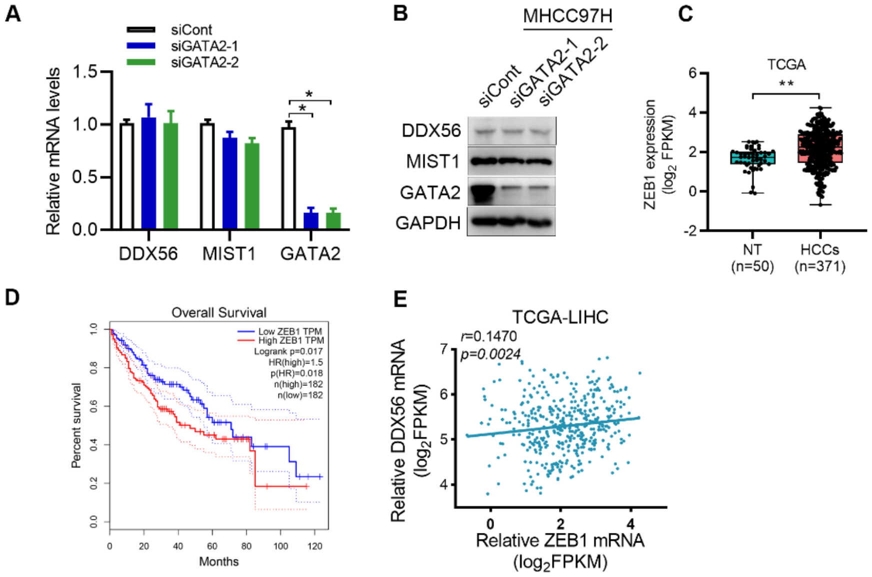
A
siCont
siGATA2-1
siGATA2-2
Relative mRNA levels
DDX56
MIST1
GATA2
B
MHCC97H
siCont
siGATA2-1
siGATA2-2
DDX56
MIST1
GATA2
GAPDH
C
TCGA
ZEB1 expression (log2 FPKM)
NT (n=50)
HCCs (n=371)
D
Overall Survival
Low ZEB1 TPM
High ZEB1 TPM
Logrank p=0.017
HR(high)=1.5
p(HR)=0.018
n(high)=182
n(low)=182
Percent survival
Months
E
TCGA-LIHC
r=0.1470
p=0.0024
Relative DDX56 mRNA (log2FPKM)
Relative ZEB1 mRNA (log2FPKM)

Figure S10 ZEB1 is the upstream regulator of DDX56 in HCC cells. (A–B) The effect of GATA2 knockdown on DDX56 and MIST1 expression was examined using RT-PCR (A) and western blot analysis (B), respectively. (C) The relative mRNA level of ZEB1 in TCGA (NT = 50, T = 371) is shown. (D) Kaplan-Meier survival analysis for HCC patients indicates that high ZEB1 expression is significantly related to shorter overall survival (* = $P < 0.05$). (E) Correlation between ZEB1 and DDX56 mRNA levels in the TCGA-LIHC database (r = 0.1470; $P = 0.0024$).

Supplementary Table S1 List of primers sequences

**Genes**	**Sequence (5'→3')**
**Real-time qPCR primers sequences**	
DDX56 F	GCCTGAAACTTCGTGACTCC
DDX56 R	GGCAAGTGACAGAGGAGACT
MIST1 F	CTCTCCAAGATCGAGACGCT
MIST1 R	CTGGACATGGTCAGGATGGT
GAPDH F	ACCCAGAAGACTGTGGATGG
GAPDH R	TCAGCTCAGGGATGACCTTG
PCNP F	CCCAACAAAGCCTACAAA
PCNP R	GCTGCTGCTACTGAAAGAG
SULT1A4 F	TGACCTGCTCATCAACACCT
SULT1A4 R	GCGGTGTGTCTTTCAGAGTC
MIS12 F	CGTGCATGCTTCGGATCTAC
MIS12 R	CTGCACTGGGCTAATGTCAC
MFSD5 F	CCTGGAAGGTCAAATTGCCA
MFSD5 R	CCACTAGGCCAAAGAGGACT
LGR4 F	AACAGTACCCAGTGAAGCCA
LGR4 R	CTGTTGTCATCCAGCCACAG
PTMA F	CTGCTAACGGGAATGCTG
PTMA R	CCTCGTCGGTCTTCTGCT
SERPINB4 F	TGAATTCAACAAATCCACTGATGC
SERPINB4 R	TGACTTTCCACCCAGGAGTT
CAB39 F	AGAGCCTCAGACAGAAGCAG
CAB39 R	TGCCCTCAAAGTCAATGAGC
DNAL1 F	CTGAGTTTGTGAAGCTGGCA

DNAL1 R	AGTTTGGGCACTCTCTTGGT
BCL2L2 F	TGGTGGCAGACTTTGTAGGT
BCL2L2 R	GGGTCTCGAACTCATCTCCA
MTAP F	AAGGTCAACTACCAGGCGAA
MTAP R	ACACTCCTCTGGCACAAGAA
HIBADH F	TTCTGGTGGTGTAGGAGCTG
HIBADH R	TTGGGCAGCAGCAAATTCAT
ST6GAL1 F	GGACCAGGCATCAAGTTCAG
ST6GAL1 R	AATGCTCTCCTTGGGCAGAT
MRPL34 F	TCAAACGCAAGAACAAGCAC
MRPL34 R	ATTCGGCGAAGGATGACCT
UPK3BL1 F	CACGGACAAACCAGGACATC
UPK3BL1 R	TGGTTGGCAGTGGGTATCAT
POLR2A F	TGTCTGCTTCTTCTGCTCCA
POLR2A R	TAGACATGTGTGAGCCGCTT
HTRA3 F	ACTTCATTGCTGACGTGGTG
HTRA3 R	CAAACAGCGGGTGTCTCAG
ENG F	CAGCAGTGTCTTCCTGCATC
ENG R	GGCTGGAATTGTAGGCCAAG
ZEB1 F	TTCACAGTGGAGAGAAGCCA
ZEB1 R	GCCTGGTGATGCTGAAAGAG
GATA2 F	GCCTGTGGCCTCTACTACAA
GATA2 R	ATCTTCCGGTTCCGAGTCTG
**MIST1 promoter sequences**	
MIST1-Promoter-204F	TTCTATCTTGGGGGCGGGGC
MIST1-Promoter+64R	AGGCTGCCCACGGGGGACAA
MIST1-Promoter+64F	GCCGCGGATCCCCAGGTAAC
MIST1-Promoter+270R	AGGTCAGAGGTCGGCAATGG
MIST1-Promoter-1974F	CAGAAGAGGTTCATGGCAAT
MIST1-Promoter+270R	AGCCGTGGAGGTCAGAGGTC
MIST1-pGL3-1974F	cgagctcttacgcgtgctagcCAGAAGAGGTTCATGGCAATAT
MIST1-pGL3+270R	tcaagggcatcggtcgacggatccAGCCGTGGAGGTCAGAGGTCGGCAA
MIST1-pGL3-1700F	cgagctcttacgcgtgctagcGCCCCTGCCCTGGTCCCTCTGGA
MIST1-pGL3-1415F	cgagctcttacgcgtgctagcCCCCTCTGAGTTCCCACCCAGAG
MIST1-pGL3--1080F	cgagctcttacgcgtgctagcCGGGAGGCTGAGGCAGGAGAATC
MIST1-pGL3-756F	cgagctcttacgcgtgctagcTCTGTGTGCTGAGCCTGAATCGGGG
MIST1-pGL3-506F	cgagctcttacgcgtgctagcTGGGGAAAAGCAGGTTGTGGGGAGG
MIST1-pGL3-204F	cgagctcttacgcgtgctagcTTCTATCTTGGGGGCGGGGCGGA
MIST1-pGL3+64F	cgagctcttacgcgtgctagcGCCGCGGATCCCCAGGTAACCC
**DDX56 promoter sequences**	

DDX56 promoter -1982F	CGAGGCGGGCGGATCACAAG
DDX56 promoter 1982R	TGCAGGAAAAACGCCACCGA
DDX56 promoter(A) -1982F	CGAGGCGGGCGGATCACAAG
DDX56 promoter(A) -1605R	GGAGGGAGAAGGCTGTTTGG
DDX56 promoter(B) -850F	TGTTTCCTTGCCTTTTCCAG
DDX56 promoter(B) -1189R	CAGTTAGCGTGTATGTATGT
pGL3-DDX56 F	cgagctcttacgcgtgctagcCCCAGCACTTTGGGAGGCCGAG
pGL3-DDX56 R	tcaagggcatcggtcgacggatccAGCGCAGCACGCACGCGCTGCA
**PTEN promoter sequences**	
PTEN promoter P1F	TGTAAACAGACTTGACAGGT
PTEN promoter P1R	TCGACCTATTCTGCGCCTTC
PTEN promoter P2F	TGTAGAGCAAGGAGTGAGTC
PTEN promoter P2R	TCTTCCTTTGCTCGGGGTGC
PTEN promoter P3F	ACTTGCCTCCGGAGCTATCA
PTEN promoter P3R	CCTTGGCCGCCGTGAAAACC
PTEN promoter P4F	GTCTCTGAGAACCGAGCTTG
PTEN promoter P4R	CTCATGGTGTCAGTCTTAGC
PTEN promoter P5F	TGATACACGCTGGCGACACA
PTEN promoter P5R	TGTGCACAGGTGAAAAGGAC
PTEN promoter P6F	GACACTGCTCAACGCACCCA
PTEN promoter P6R	TAGGAGGGGGCAGAGCGGTA
PTEN promoter P7F	GCCCTGCCCTCCCCTCGCCC
PTEN promoter P7R	TGAGCGCAGTCGCGTCCCAG
**Biotin-labeled DNA probes from the MIST1 promoters**	
MIST1 F Biotin-204~-161F	TTCTATCTTGGGGGCGGGGCGGAAGCTGGGGGCGGGGCTGCGCG
MIST1 R Biotin-204~-161R	CGCGCAGCCCCGCCCCCAGCTTCCGCCCCGCCCCCAAGATAGAA
MIST1 F Biotin-160~-117F	GCCACCTGGTCCGGGAGCCCCAGGCGCCGGCCTCCCGCCCGCCC
MIST1 R Biotin-160~-117R	GGGCGGGCGGGAGGCCGGCGCCTGGGGCTCCCGGACCAGGTGGC

MIST1 F Biotin-116~-73F	GCCCGCAGGTGCCGCCACTGCTGCCCAGCGCCCGCCGGGACACC
MIST1 R Biotin-116~-73R	GGTGTCCCGGCGGGCGCTGGGCAGCAGTGGCGGCACCTGCGGGC
MIST1 F Biotin-72~-29F	CGGACCCCGGCCCCGCCCCGGCCCCGCCCCGAGGGCTCATTTGC
MIST1 R Biotin-72~-29R	GCAAATGAGCCCTCGGGGCGGGGCCGGGGCGGGGCCGGGGTCCG
MIST1 F Biotin-28~+15F	ATGCGTCCGCGGCCCCTCCCGGGGCGGCTAAAGCGACGTGTCCT
MIST1 R Biotin-28~+15R	AGGACACGTCGCTTTAGCCGCCCCGGGAGGGGCCGCGGACGCAT
MIST1 F Biotin+16~+64F	TGTCCCCCGTGGGCAGCCTCGGCGCGTGTGGCCGCGGATCCCCAGGTAAC
MIST1 F Biotin+16~+64R	GTTACCTGGGGATCCGCGGCCACACGCGCCGAGGCTGCCCACGGGGGACA

Supplementary Table S2 Antibody used in this study

Antibody	Company	Cat.NO	Application
DDX56	GeneTex	GTX115551	WB,IHC,IF
	Santa	sc-393078	IP,ChIP
	Origene	UM800049	EMSA
MIST1	CST	#14896	WB,IHC
	Novus	NBP2-22478	IF,IHC
	Proteintech	25140-1-AP	IP
MECOM	CST	#2593	WB,IF,IP,ChIP
PTEN	Proteintech	22034-1-AP	WB
SNAI1	Proteintech	13099-1-AP	WB
p-AKT	Proteintech	28731-1-AP	WB
GAPDH	Proteintech	60004-1-Ig	WB
H3K9me1	CST	#14186	ChIP
ZEB1	Proteintech	21544-1-AP	WB,ChIP
GATA2	Proteintech	11103-1-AP	WB

Supplementary Table S3 shRNA sequences used in this research.

Gene	Sequences
shDDX56-1 sense	5'-CACCGTGTCTGCTGTGCTCAACTTTGCGAACAAAGTTGAGCACAGCAGACA-3'
shDDX56-1 anti-sense	5'-AAAATGTCTGCTGTGCTCAACTTTGTTCGCAAAGTTGAGCACAGCAGACAC-3'
shDDX56-2 sense	5'-CACCATTGTGGAGCACAGGCTTACACGAATGTAAGCCTGTGCTCCACAA-3'
shDDX56-2 anti-sense	5'-AAAATTGTGGAGCACAGGCTTACATTCGTGTAAGCCTGTGCTCCACAAT-3'
shMIST1-1 sense	5'-CACCGCGGATGCACAAGCTAAATAACGAATTATTTAGCTTGTGCATCCGC-3'
shMIST1-1 anti-sense	5'-AAAAGCGGATGCACAAGCTAAATAATTCGTTATTTAGCTTGTGCATCCGC-3'
shMIST1-2 sense	5'-CACCGCAAATCGCTGACGGCCACCATCGAAATGGTGGCCGTCAGCGATTTG-3'
shMIST1-2 anti-sense	5'-AAAACAAATCGCTGACGGCCACCATTTCGATGGTGGCCGTCAGCGATTTGC-3'
shMECOM-1 sense	5'-CACCGCCGTTACACAGAAAGTCCAACGAATTGGACTTTCTGTGTAACGGC-3'
shMECOM-1 anti-sense	5'-AAAAGCCGTTACACAGAAAGTCCAATTCGTTGGACTTTCTGTGTAACGGC-3'
shMECOM-2 sense	5'-CACCGCACTACGTCTTCCTTAAATACGAATATTTAAGGAAGACGTAGTGC-3'
shMECOM-2 anti-sense	5'-AAAAGCACTACGTCTTCCTTAAATATTCGTATTTAAGGAAGACGTAGTGC-3'
siZEB1-1	ATAGAGGCTACAAGCGCTTTA
siZEB1-2	GCTGTTGTTCTGCCAACAGTT
siGATA2-1	GTGCAAATTGTCAGACGACAA
siGATA2-2	CCGGCACCTGTTGTGCAAATT

Supplementary Table S4 Correlation between DDX56 expression and clinical characteristics of 72 HCC patients (Cohort I)

**Clinicopathologic parameters**	**No. of specimens**	**DDX56 Low**	**DDX56 High**	***P* Value**
Sex				
Female	7	5	2	0.0947
Male	65	22	43	
Antigen (CEA)				
≤5 ng/mL	63	12	51	0.3826
>5 ng/mL	9	3	6	
α-fetoprotein (AFP)				
≤20 ng/mL	28	11	17	**0.0486**
>20 ng/mL	44	7	37	
Cirrhosis				
Yes	54	13	41	0.7603
No	18	5	13	
Tumor size				
≤3cm	18	7	11	**0.0341**
>3 cm	54	7	47	
Multiple tumor				
Yes	20	12	8	0.578

No	52	36	16	
Tumor grade				
1	18	1	17	0.061
2	45	11	34	
3	9	4	5	
Vascular invasion				
Yes	22	6	16	0.2019
No	50	22	28	
HBV				
Yes	63	9	54	**0.0496**
No	9	4	5	

*Pearson chi-square test.$P<0.05$.The bold values mean the clinical variable with statistically significance.

Supplementary Table S5 Clinical information for samples from patients with HCC used in this study

Patient NO.	Age	Gender	Tumor Size （cm）	Tumor number	Diagnosis	Tumor grade	Vascular Invasion	Liver cirrhosis
1	55	Male	5.5	1	Hepatocellular carcinoma	II	No	No
2	65	Male	4	1	Hepatocellular carcinoma	II	No	Yes
3	77	Male	4.5	1	Hepatocellular carcinoma	I	No	Yes
4	81	Male	10	≥2	Hepatocellular carcinoma	II	Yes	No
5	69	Male	8.5	1	Hepatocellular carcinoma	II	No	Yes
6	68	Male	6	1	Hepatocellular carcinoma	II	No	No
7	74	Male	3	≥2	Hepatocellular carcinoma	III	Yes	No
8	52	Male	2.8	1	Hepatocellular carcinoma	I	No	No
9	72	Male	7	1	Hepatocellular carcinoma	II	Yes	Yes
10	59	Male	11	1	Hepatocellular carcinoma	II	No	Yes
11	70	Male	6	1	Hepatocellular carcinoma	II	No	Yes
12	53	Male	2	1	Hepatocellular carcinoma	II	No	Yes
13	73	Male	11	1	Hepatocellular carcinoma	III	No	Yes
14	68	Male	10	1	Hepatocellular carcinoma	II	Yes	Yes
15	80	Male	16	1	Hepatocellular carcinoma	II	Yes	Yes
16	60	Male	10	1	Hepatocellular carcinoma	II	No	Yes
17	75	Male	4	≥2	Hepatocellular carcinoma	II	No	Yes
18	67	Male	10	≥2	Hepatocellular carcinoma	II	Yes	Yes
19	58	Male	2.8	≥2	Hepatocellular carcinoma	I	Yes	Yes
20	60	Male	9	1	Hepatocellular carcinoma	II	No	No
21	60	Male	4	≥2	Hepatocellular carcinoma	II	Yes	No
22	78	Male	4.5	1	Hepatocellular carcinoma	III	Yes	Yes
23	65	Male	5.5	≥2	Hepatocellular carcinoma	II	Yes	Yes
24	41	Female	8	1	Hepatocellular carcinoma	III	No	No
25	58	Male	3	≥2	Hepatocellular carcinoma	III	No	No
26	69	Male	6	1	Hepatocellular carcinoma	II	Yes	No
27	43	Male	6	1	Hepatocellular carcinoma	II	No	Yes
28	51	Male	7	1	Hepatocellular carcinoma	I	Yes	Yes
29	55	Male	12	≥2	Hepatocellular carcinoma	II	Yes	No
30	30	Male	9	1	Hepatocellular carcinoma	II	Yes	Yes
31	60	Male	4	1	Hepatocellular carcinoma	II	No	Yes
32	52	Female	4	1	Hepatocellular carcinoma	I	Yes	Yes
33	72	Male	8	1	Hepatocellular carcinoma	III	No	Yes
34	26	Female	2.8	≥2	Hepatocellular carcinoma	II	No	Yes
35	68	Male	4.5	1	Hepatocellular carcinoma	II	No	Yes
36	56	Male	4	1	Hepatocellular carcinoma	I	Yes	Yes

37	65	Male	11	≥2	Hepatocellular carcinoma	III	No	Yes
38	22	Female	6	1	Hepatocellular carcinoma	I	No	Yes
39	49	Male	7.5	1	Hepatocellular carcinoma	II	No	Yes
40	48	Male	6	1	Hepatocellular carcinoma	II	No	Yes
41	74	Male	3	≥2	Hepatocellular carcinoma	II	No	Yes
42	46	Male	4.5	≥2	Hepatocellular carcinoma	I	No	No
43	42	Male	6	1	Hepatocellular carcinoma	II	No	Yes
44	65	Male	5	1	Hepatocellular carcinoma	II	No	Yes
45	43	Male	2	≥2	Hepatocellular carcinoma	II	No	Yes
46	52	Male	6	1	Hepatocellular carcinoma	II	No	Yes
47	49	Male	4.5	1	Hepatocellular carcinoma	II	No	Yes
48	42	Male	4.9	1	Hepatocellular carcinoma	II	No	Yes
49	60	Male	4	≥2	Hepatocellular carcinoma	III	No	Yes
50	60	Male	8.6	≥2	Hepatocellular carcinoma	III	No	Yes
51	50	Male	9	1	Hepatocellular carcinoma	II	No	Yes
52	52	Male	11	1	Hepatocellular carcinoma	II	No	Yes
53	42	Male	11.5	≥2	Hepatocellular carcinoma	II	Yes	Yes
54	39	Male	2.8	1	Hepatocellular carcinoma	I	No	Yes
55	27	Male	12	≥2	Hepatocellular carcinoma	II	Yes	Yes
56	19	Male	1.8	1	Hepatocellular carcinoma	I	No	Yes
57	64	Male	4	1	Hepatocellular carcinoma	II	Yes	Yes
58	45	Female	9	1	Hepatocellular carcinoma	II	Yes	Yes
59	39	Male	2	1	Hepatocellular carcinoma	II	No	Yes
60	42	Male	6	1	Hepatocellular carcinoma	I	No	No
61	68	Female	2.5	1	Hepatocellular carcinoma	I	No	Yes
62	48	Male	6	1	Hepatocellular carcinoma	I	No	Yes
63	51	Male	8	1	Hepatocellular carcinoma	I	Yes	Yes
64	63	Male	5	1	Hepatocellular carcinoma	I	No	No
65	42	Male	2.3	≥2	Hepatocellular carcinoma	II	No	Yes
66	70	Male	2.5	1	Hepatocellular carcinoma	II	No	Yes
67	67	Male	3	1	Hepatocellular carcinoma	I	No	Yes
68	41	Male	2.6	1	Hepatocellular carcinoma	I	No	No
69	48	Male	8.5	1	Hepatocellular carcinoma	II	No	No
70	57	Male	14	≥2	Hepatocellular carcinoma	II	Yes	No
71	50	Female	2.8	1	Hepatocellular carcinoma	II	No	Yes
72	62	Male	2	1	Hepatocellular carcinoma	I	No	No

Supplementary Table S6 Association of DDX56 with clinical characteristics of 32 HCC patients (Cohort II) for immunohistochemistry.

**Clinicopathologic parameters**	**No. of specimens**	**DDX56 Low**	**DDX56 High**	***P* Value**
Sex				
Female	7	1	6	0.2117
Male	25	11	14	
Age (years)				
≤55	19	8	11	0.7128
>55	13	4	9	
Tumor differentiation				
well	9	4	5	0.7643
moderately	16	5	11	
poorly	7	3	4	
Tumor size				
≤5cm	22	5	17	**0.0184***
>5 cm	10	7	3	
Liver cirrhosis				
Without	19	6	13	0.4735
With	13	6	7	
HBV infection				
Negative	9	2	7	0.4224

Positive	23	10	13

*Pearson chi-square test.*P*<0.05. Abbreviations: AFP, alpha-fetoprotein; CEA, Antigen;

Supplementary Table S7 The transcriptional regulators bound with DDX56 were identified in IP-MS

Protein ID	Accession	Description
2156	**sp|Q03112|MECOM_HUMAN**	**Histone-lysine N-methyltransferase MECOM OS=Homo sapiens OX=9606 GN=MECOM PE=1 SV=3**
2824	sp|P57078|RIPK4_HUMAN	Receptor-interacting serine/threonine-protein kinase 4 OS=Homo sapiens OX=9606 GN=RIPK4 PE=1 SV=1
1516	sp|Q8IWS0|PHF6_HUMAN	PHD finger protein 6 OS=Homo sapiens OX=9606 GN=PHF6 PE=1 SV=1
1652	sp|Q9H7Z3|NRDE2_HUMAN	Nuclear exosome regulator NRDE2 OS=Homo sapiens OX=9606 GN=NRDE2 PE=1 SV=3
2395	sp|Q92908|GATA6_HUMAN	Transcription factor GATA-6 OS=Homo sapiens OX=9606 GN=GATA6 PE=1 SV=2
